# Improving the performance of Bayesian phylogenetic inference under relaxed clock models

**DOI:** 10.1186/s12862-020-01609-4

**Published:** 2020-05-14

**Authors:** Rong Zhang, Alexei Drummond

**Affiliations:** 1 School of Computer Science, University of Auckland, Auckland, New Zealand; 2 School of Biological Sciences, University of Auckland, Auckland, New Zealand

**Keywords:** Bayesian MCMC, Bayesian phylogenetics, Proposal kernel, Genetic distances, Divergence times, Evolutionary rates

## Abstract

**Background:**

Bayesian MCMC has become a common approach for phylogenetic inference. But the growing size of molecular sequence data sets has created a pressing need to improve the computational efficiency of Bayesian phylogenetic inference algorithms.

**Results:**

This paper develops a new algorithm to improve the efficiency of Bayesian phylogenetic inference for models that include a per-branch rate parameter. In a Markov chain Monte Carlo algorithm, the presented proposal kernel changes evolutionary rates and divergence times at the same time, under the constraint that the implied genetic distances remain constant. Specifically, the proposal operates on the divergence time of an internal node and the three adjacent branch rates. For the root of a phylogenetic tree, there are three strategies discussed, named Simple Distance, Small Pulley and Big Pulley. Note that Big Pulley is able to change the tree topology, which enables the operator to sample all the possible rooted trees consistent with the implied unrooted tree. To validate its effectiveness, a series of experiments have been performed by implementing the proposed operator in the BEAST2 software.

**Conclusions:**

The results demonstrate that the proposed operator is able to improve the performance by giving better estimates for a given chain length and by using less running time for a given level of accuracy. Measured by effective samples per hour, use of the proposed operator results in overall mixing more efficient than the current operators in BEAST2. Especially for large data sets, the improvement is up to half an order of magnitude.

## Background

Bayesian phylogenetics puts an emphasis on estimating a probability distribution over parameters of interest, including the phylogenetic tree topology and divergence times, given the data. The Metropolis-Hastings Markov chain Monte Carlo (MCMC) [1, 2] algorithm has been the primary computational tool used in Bayesian phylogenetics for sampling from the posterior distribution. This paper is aimed at improving the performance of the relaxed clock model in Bayesian phylogenetic analysis.

Early implementations of Bayesian phylogenetic inference assumed a strict molecular clock where the evolutionary rates are the same at every branch [3]. This was the preferred method for estimating divergence times [4, 5]. The introduction of relaxed molecular clocks allowed for the estimation of divergence times [6] and phylogeny [7] in the presence of rate heterogeneity among branches. Since then, the relaxed clock model has been widely applied, such as the study of Nothofagus [8] and flowering plants [9]. Many aspects of the performance and accuracy of relaxed clock models have subsequently been investigated (e.g. [10, 11]).

Bayesian phylogenetic inference via MCMC is computationally intensive for large data sets. Two approaches to improve efficiency are (i) by making faster likelihood calculations, and (ii) by incorporating more effective proposal kernels. Calculating the phylogenetic likelihood is computationally expensive. Hence, researchers have tried many ways to tackle the computation burden in the likelihood calculations, such as detection of repeating sites [12], approximate methods (e.g. [13, 14]) and the use of parallelisation strategies (e.g. BEAGLE [15]).

However the overall efficiency of the sampling process also depends strongly on the construction of the proposal mechanism. An effective proposal mechanism is proficient at exploring the posterior distribution, and can do so with fewer steps in the MCMC chain. Therefore fewer likelihood calculations are required, since each step in the chain that changes the tree or substitution parameters requires a likelihood calculation.

A major limitation in Bayesian MCMC analysis of phylogeny lies in the efficiency with which operators sample the tree space [16, 17]. Fast and reliable estimation is dependent on a good mixture of operators, since the posterior distribution often exhibits correlations between the tree and other random variables.

In this paper, we present a novel operator that works alongside standard operators by proposing moves within a subspace of constant genetic distances. Namely, the proposed operator changes both divergence times of nodes and neighbouring branch rates so that the implied genetic distances are not changed. For time-reversible substitution models the phylogenetic likelihood will also be unchanged under this operation. The proposed operator has been implemented and tested in BEAST2 [18].

## Preliminaries

### Bayesian MCMC

Let **D**, *g* and ***Φ*** denote the data, phylogenetic time-tree and a set of evolutionary parameters respectively. The time-tree *g*={*E*,**t**} consists of a directed edge graph, *E*, defining a rooted tree topology on a set of labelled taxa and a set of associated divergence times **t** (for details see e.g. [19]). The posterior probability density can be calculated using Eq. 1. It consists of prior distributions for the tree and the parameters, a phylogenetic likelihood that conveys information from data, and the posterior distribution to be inferred. These are denoted in the form of probability densities by *p*(*g*),*p*(**Φ**),*p*(**D**|*g*,***Φ***),*p*(*g*,***Φ***|**D**) respectively. From a Bayesian perspective, the phylogenetic trees and the parameters are random variables described by a posterior probability distribution given the observed data **D**.

1 $$ p(g,\mathbf{\Phi} |\mathbf{D}) = \frac{{p (\mathbf{D}|g,\mathbf{\Phi}) p (g) p (\mathbf{\Phi})}}{{p (\mathbf{D})}}  $$

However, due to the state space being high dimensional and the marginal likelihood being infeasible to calculate, MCMC is adopted to sample the posterior distribution. Specifically, MCMC algorithms construct a Markov chain whose stationary distribution is the posterior distribution *p*(*g*,**Φ**|**D**), in such a way that the computation of the marginal likelihood *p*(**D**) is avoided.

### Tree proposals

We use the term “operator" to describe an algorithm that can be used to draw a new state *θ*^′^ given an existing state *θ*={*g*,**Φ**} from a specific proposal kernel *q*(*θ*^′^|*θ*) and also return the Hastings-Green ratio for the proposed state transition [2, 20].

Standard naïve operators such as the random walk operator propose the new state *θ*^′^ by adding a random variate to a component of the current state *θ* [21]. Similarly, scale operators multiply a subset of the current state by a random scale factor [22]. They are suitable for working on a single random variable, or a single component of the model, for example the population size parameter of the coalescent tree prior. Standard operators for the tree topology and divergence times include the subtree slide operator, Wilson-balding and narrow exchange operators [19, 23].

In this paper, the novelty of the proposed operators lies in maintaining the genetic distance *d* while changing the rate *r* and divergence time *t*. The reason is that the likelihood along one branch is constant if its distance is fixed, i.e. *d*=*r*×*t*, noting that the likelihood is calculated based on transition probability matrix for each branch of $\phantom {\dot {i}\!}{e^{\mathbf {Q}d_{i}}}$, where *d*_*i*_ is the branch length in units of substitutions per site for branch *i*. In this way, the joint distribution on rates and divergence times can be explored without proposing states that would adversely affect the phylogenetic likelihood.


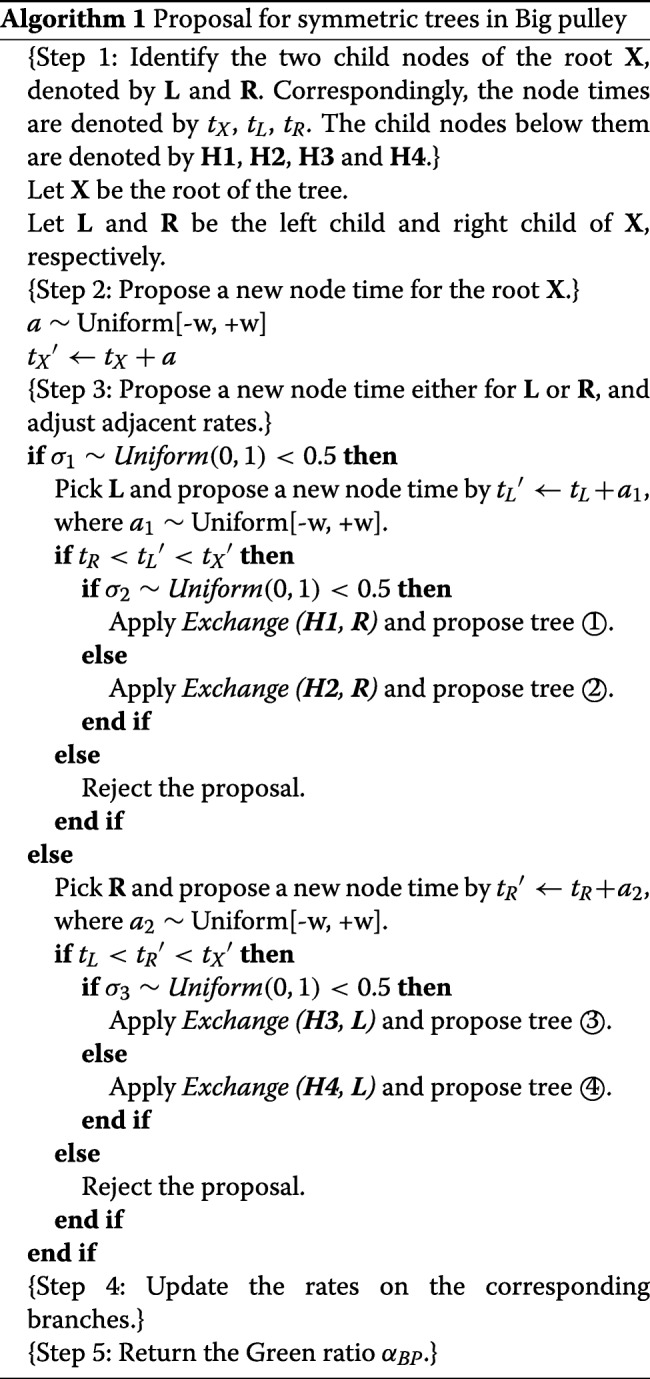


### Uncorrelated relaxed clock model

Molecular clocks model how molecular sequences evolve along branches in the phylogenetic tree, so that a time tree can be reconciled with the genetic distances between sequences. In this paper, uncorrelated relaxed clock models are adopted, where the rates are drawn independently and identically from a given prior distribution, such as the log-normal distribution [7]. As a result, the rates can vary markedly between parent and child branches.

Referring to the Bayesian framework in Eq. (1), the joint inference of evolutionary rates **r** and the time tree *g* can be obtained by the conditional distribution in Eq. 2:

2 $$ p(g,\mathbf{r},\boldsymbol{\Phi} |\mathbf{D}) = \frac{{p(\mathbf{D}|g,\mathbf{r},\mathbf{\Phi})p(\mathbf{r})p(g)p(\mathbf{\Phi})}}{{p(\mathbf{D})}} \text{,}  $$

where *p*(**r**) is the prior for rates specified in uncorrelated relaxed clock model. In the constructed Markov chain, the operator proposes a new state *θ*^′^=(**r**^′^,*g*^′^,**Φ**^′^), from the original state *θ*={**r**,*g*,**Φ**}.

While the proposed operator is introduced based on uncorrelated clock models, it could equally be applied to any other relaxed clock that applies a rate parameter to each branch, such as autocorrelated clock models [6].

## Results

To validate the correctness and determine the efficiency, we conducted a series of experiments by implementing the Constant Distance operator in BEAST2 [18].

First, we perform a well-calibrated simulation study, which tests our operator alongside existing operators. Correctness was further confirmed by sampling trees from the prior distribution i.e. without data (Refer to Appendix 2 section for more details). By comparing effective sample sizes (ESS) [24] and running times, it is demonstrated that the performance is improved when including our proposed operator. Finally, the posterior correlation of rates and node times are discussed.

### Well-calibrated simulation study

A well-calibrated simulation study is a powerful tool for evaluating and validating the implementation of a Bayesian model [25].

**Fig. 1:**
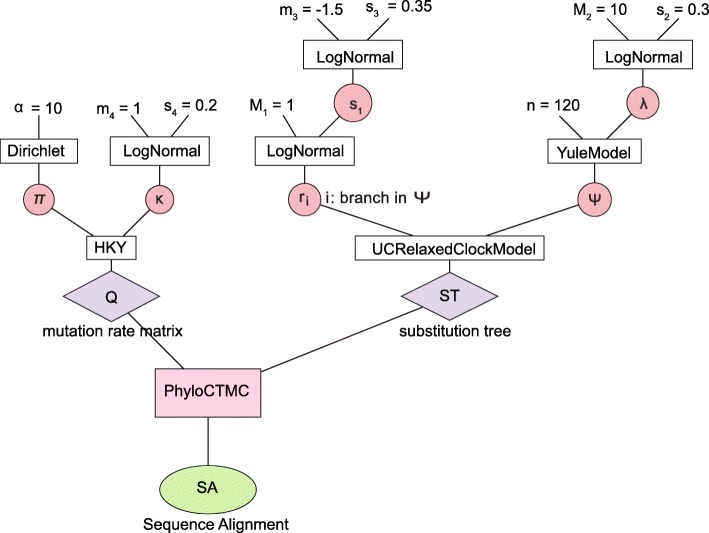
The models and prior distributions to simulate the sequence data. The sequence alignment (SA) is simulated through a phylogenetic continuous-time Markov Chain (PhyloCTMC) that consists of a substitution model (HKY) and an uncorrelated relaxed clock model (UCRelaxedClockModel). The random variables in HKY model construct the mutation rate matrix (*Q*), including base frequencies (**π**={*π*_*A*_,*π*_*C*_,*π*_*G*_,*π*_*T*_}) and kappa (*κ*). The time trees (*ψ*) and branch rates (*r*_*i*_ for each branch *i* in *ψ*) construct the substitution tree (ST). The branch rates have a LogNormal prior with fixed mean 1 and certain standard deviation (denoted by *s*_1_). And the time trees have a Yule model prior with birth rate (*λ*) having a LogNormal prior. The other prior distributions include a Dirichlet distributions on **π**, a LogNormal distribution on *κ*, and a LogNormal distribution on *s*_1_. For notations in LogNormal distributions, the uppercase letters represent the parameters in real space, and the lowercase letters represent the parameters in log space. In all the simulations, the number of taxa is fixed at 120 (n = 120)

Figure 1 shows the Bayesian model used in this study, which includes the evolutionary model and the prior distributions of parameters. As is shown in the figure, the sequence alignment is simulated by a phylogenetic continuous-time Markov chain in BEAST2. It contains a substitution rate matrix given by the HKY85 [26] model and a substitution tree determined by an uncorrelated relaxed clock model and Yule model. More specifically, base frequencies ***π*** follow a Dirichlet distribution and the transition-transversion ratio *κ* follows a log-normal prior distribution. The distribution of node times is described in a Yule tree *ψ* with hyperparameter birth rate *λ* following a log-normal distribution. The rates *r*_*i*_ follow a log-normal distribution with mean of 1 and standard deviation *s*_1_ following a hyperprior distribution.

**Fig. 2:**
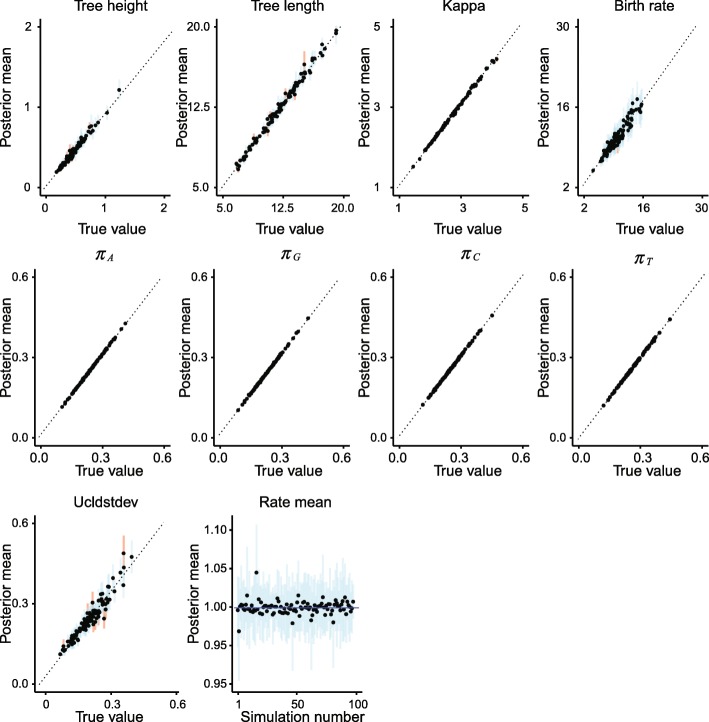
Well-calibrated simulation study with 120 taxa. Each point is a separate simulated dataset

First, we sampled parameters and trees from the full model 100 times. The random parameters included: standard deviation of rates across branches *s*_1_, birth rate *λ*, base frequencies ***π*** and transition-transversion bias *κ*. Second, we simulated nucleotide alignments using the simulated parameters. In total, 100 data sets were simulated, each with 120 taxa. Third, we used BEAST2 with the Constant Distance operator to infer the tree and parameters from each of the 100 simulated data sets in turn. Finally, the posterior estimates of the parameters were compared with the real values that were used to simulate the corresponding sequence alignment. The comparisons are shown in Fig. 2.

These results show that the true values of the parameters are within the 95% highest posterior density (HPD) interval approximately 95% of the time (Table 1). This well-calibrated simulation study formed part of the validation of our implementation of the Constant Distance operator.

**Table 1:** Percentage of real values lying in the 95% HPD in Fig. 2

Parameters	Coverage	Parameters	Coverage
Tree height	89	Ucldstdev	91
Tree length	91	*π*_*A*_	94
Kappa	97	*π*_*C*_	96
Birth rate	99	*π*_*G*_	95
Rate mean	100	*π*_*T*_	97

### Performance comparison

To evaluate the performance of Constant Distance operator in a Bayesian phylogenetic analysis, we explored the time required to adequately sample the posterior distribution. This was achieved by examining (i) the total time taken by BEAST2 to complete the MCMC inference (running time), and (ii) the effective sample size (ESS) of the sampled parameters. The effective sample size of a parameter is the number of effectively independent samples from the posterior distribution. Larger ESS indicates a better approximation of the marginal posterior distribution of the parameter. We used Tracer [24] to compute ESS.

For each dataset, we compared two operator configurations. 1) Using the current operators in BEAST2 to sample discrete rate categories (Category). 2) Using the Constant Distance operator to sample continuous rates specified by an uncorrelated related clock model (Cons). The Category configuration is the default setting in BEAST 2.5. We aim to compare the performance of the Constant Distance operator to that of the existing operator schedule. In each configuration, the data set was analyzed 20 times with the prior distributions and all other model specifications held constant. The details of operator weights used are given in Appendix 3.1. Each setting is benchmarked using an Intel(R) Xeon(R) Gold 6138 CPU (2.00 GHz).

We performed the analysis on two sets of simulated sequence alignment (See Appendix 3.2 for more details). The simulated data sets both have 20 taxa but different sequence lengths, i.e. one data set containing 500 sites, the other containing 1,000 sites. Moreover, we used four real data sets to further evaluate the performance of Constant Distance operator, including a primate data set [27] and three other data sets (Anolis [28], RSV2 [29, 30] and HIV-1 [31]) in BEAST2 [32].

**Fig. 3:**
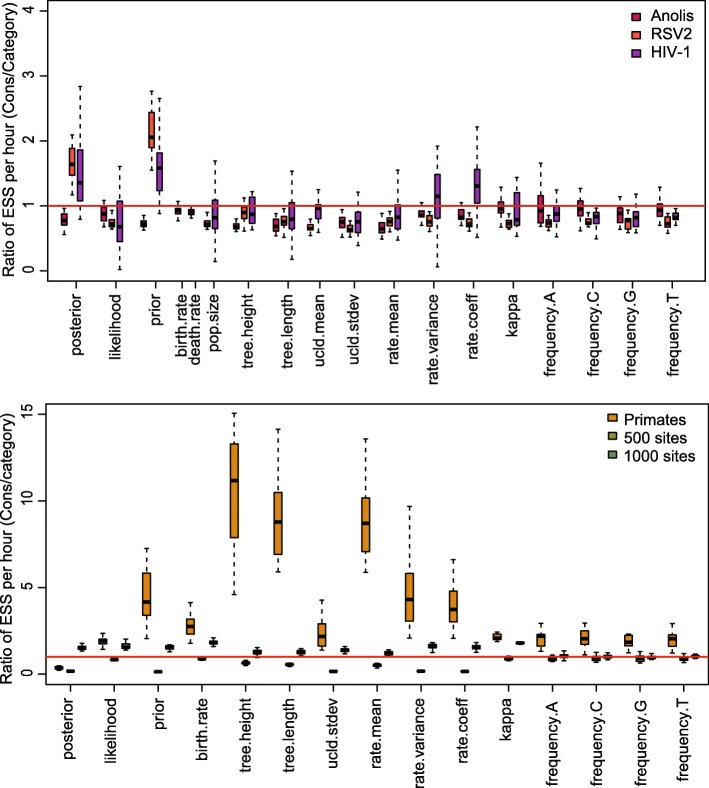
Comparison of ESS and running time. There are 6 data sets analysed, including 4 real data sets and 2 simulated data sets with different number of sites, as is shown in the legend. The red line represent the position where the ratio of ESS per hour is equal to 1. The horizontal axis represents the names of sampled parameters

The ESS and running time are summarised in Fig. 3 and Table 2. To be more specific, we measure the efficiency by ESS per hour, which is calculated by the ESS of parameters in one simulation divided by the running time in hours. Then we compare the efficiency of two configurations by calculating the ratios of ESS per hour for simulations in the two configurations. If the ratio is larger than 1, then ESS per hour of Cons configuration is larger than that of Category configuration. As is shown in Fig. 3, the efficiency varies in different data sets and also depends on what model is used in the analysis. For Anolis data set (29 taxa and 1456 sites), Category configuration performs better than Cons configuration, since most ratios of the parameters are slightly below the red line (which means smaller than 1). Moreover, for RSV2 (129 taxa and 629 sites) and HIV-1 data sets (117 taxa and 663 sites), some ratios of the parameters, “posterior” and “prior” in particular, are above the red line (larger than 1), which indicates that Cons configuration provides larger ESS per hour. Although there are several parameters sampled by Cons configuration having smaller ESS per hour, it should be noticed that the ratio is calculated by choosing random simulations in the two configurations (See Appendix 3.3 for more details). Additionally, it is worth noting that the efficiency is improved more obviously in simulated data set having 1000 sites, compared with the data sets having 500 sites. This indicates that the proposed operators behave better when sequence length is long. More specifically, in Primates data set (87 taxa and 19220 sites), the longer molecular sequence provides more accurate genetic distances, which leads to peaked likelihood distributions. In this circumstance, the proposed operators sample rates and node times that fit the constant genetic distances more efficiently.

**Table 2:** Summary of ESS and running time

Data	Configuration	Average running	Parameter	ESS
		time (hour)		
Anolis	Category	0.3788	frequency.A	698.95
	Cons	0.4212		750.53
RSV2	Category	2.0509	prior	1231.32
	Cons	2.6742		3409.88
HIV-1	Category	2.6040	prior	387.83
	Cons	3.2680		753.48
Primates	Category	31.4059	rate.mean	71.79
	Cons	21.6584		422.24
Simulated data	Category	0.0728	frequency.G	819.39
with 500 sites	Cons	0.0834		837.50
Simulated data	Category	0.4403	frequency.G	2760.41
with 1000 sites	Cons	0.4863		2961.67

Table 2 lists the average running time of 20 simulations for each data set. It can be seen that Cons configuration finished simulations with a little bit more time in most cases. This is because the continuous rates have to be adjusted for a new clock standard deviation (See Appendix Section 4 for more details). Moreover, Table 2 also shows the parameter that has the smallest ESS in Category configuration, and is compare with the corresponding ESS in Cons configuration. Although the improvement in ESS is not obvious for both simulate data sets, it is noticed that ESS of the parameters are much larger in Cons configuration for all the real data sets. After calculating the ESS per hour, we conclude that Cons configuration improved the efficiency of the worst estimated parameter in Category configuration by a factor of 1.55 to 8.53.

### Correlation analysis of rates and branch lengths

In this section, we conduct a pairwise comparison between rates and branch lengths in units of time. We used a data set of ratite mitochondrial genomes [33]. This data set includes 7 species of ratites and an alignment of 10767 sites. After analysing the ratites data set in BEAST2 using the Constant Distance operator, we calculated the Pearson coefficient between the rates and the times across branches to investigate the posterior correlation of these parameters.

**Fig. 4:**
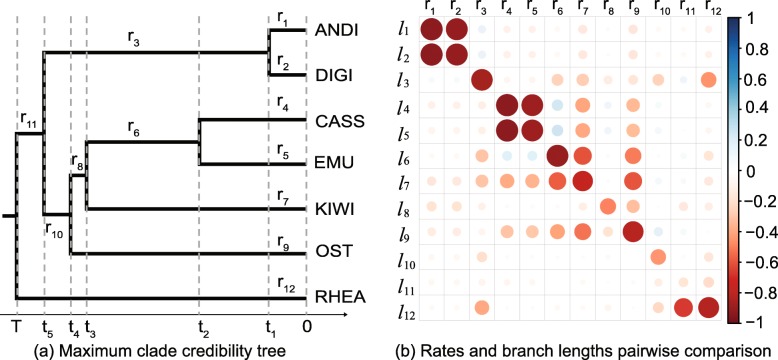
Correlation analysis in the ratites tree. *l* represents the length of a branch, that is the time difference between a parent node and a child node, where *l*_1_=*l*_2_=*t*_1_−0,*l*_3_=*l*_4_=*t*_2_−0,*l*_5_=*t*_3_−0,*l*_6_=*t*_4_−0,*l*_7_=*T*−0,*l*_8_=*t*_5_−*t*_1_,*l*_9_=*t*_3_−*t*_2_,*l*_10_=*t*_4_−*t*_3_,*l*_11_=*t*_5_−*t*_4_ and *l*_12_=*T*−*t*_5_. The rates and branch lengths are converted into log space and then Pearson’s coefficients are computed, which range from -1 to 1. Blue indicates positive correlations and red indicates negative correlations. The darker the colour, the stronger the correlation

The results are summarised in Fig. 4. Figure 4a presents the topology of the maximum clade credibility tree. We utilised the programme TreeStat2 [34] to obtain the filtered trees that have the same topology as the maximum clade credibility tree from the sampled trees in MCMC chain. This means the trees that have different shared common ancestors of each taxon from the reference tree are filtered out.

Afterwards, Fig. 4b shows the pairwise comparison of the 12 branch rates and 12 branch lengths (in time) on these filtered trees. As can be seen from the diagonal, the rate on one branch is negatively correlated with the length of that branch, which indicates that an older divergence time will lead to a smaller rate. This is because the primary signal in the data is genetic distance, so that there will be a range of rates and divergence times that are consistent with the genetic distances, but the products of these quantities will vary less than the individual parameters. The consequence is that there will tend to be a negative relationship between rate *r*_*i*_ and branch length *l*_*i*_ i.e. *r*_*i*_=*d*_*i*_/*l*_*i*_. At the same time, there will tend to be a positive relationship between rate *r*_*i*_ and its parent’s branch length *l*_*ip*_, since a larger *l*_*ip*_ leads to a smaller *l*_*i*_. Moreover, for cherries that share the same branch length in the tree, they will tend to have the same correlation pattern. Take ANDI and DIGI as an example. *r*_1_ and *l*_1_ are negatively correlated, but *r*_1_ and *l*_8_ are positively correlated, which is also the correlation of *r*_2_,*l*_2_ and *l*_8_.

It is precisely this form of correlation structure in the posterior that our operator anticipates, and these correlations are the reason that our operator performs better than naive alternatives.

### Sampling a fixed unrooted tree

A limiting case for the relaxed molecular clock model (and one exploited in some of our validation tests) occurs for long sequences, when the branch lengths of the unrooted tree, in units of expected substitutions per site, become known without error. With full length genomes now available, although inferring trees from genomes involves complexities and assumptions such as a good partition scheme [35], this limiting case might be approached in some data sets. As a simple test in this paper, this gives rise to an alternative approach to analysis, where divergence times, a root position and the branch rates are random variables, and the data are a set of branch lengths in units of substitution on a known unrooted tree topology.

Previous work done by Reis and Yang [14] also tried to approximate the likelihood of such an unrooted tree in Bayesian phylogenetic inference. Similar researches in [6, 13] show that these methods can account for rate changes in a relaxed clock model, but the genetic distances are not fixed, for example Stéphane Guindon used a Gibbs sampling algorithm [13]. Outside of the Bayesian MCMC formalism, least-squares criteria [36] and maximum likelihood [37, 38], can also be applied to estimate substitution rates and divergence times in unrooted trees.

In this section, we investigated this approach on a fixed substitution tree reconstructed from whole mitochondrial genomes from a set of ratite species [33]. Since no uncertainty is admitted in the genetic distances and the proposed operator doesn’t change the genetic distances, the phylogenetic likelihood is no longer needed and the unrooted tree becomes the data, rather than a multiple sequence alignment.

**Fig. 5:**
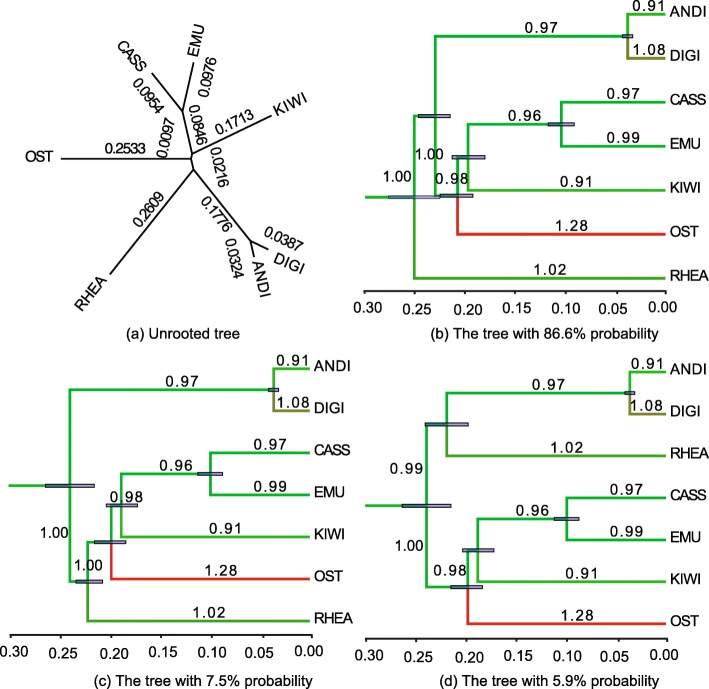
Illustration of sampling a fixed unrooted tree. In subfigure (**a**), the unrooted tree is obtained from the ratites data set [33] by a maximum likelihood method [39] and the labeled numbers represent genetic distances. The three unique tree topologies in (**b**) (**c**) and (**d**) are obtained from the sampled trees by using program TreeTraceAnalysis [41]. The branch rates and node times are summarised by using program TreeAnnotator [42]. The labeled numbers represent the posterior mean of rates on the corresponding branches. The colour of branches from green to red indicates the rates increasing from small to large, and the blue bars represent the 95% HPD of the corresponding node times

First of all, we used the ratites data set to construct an unrooted tree with PhyML 3.0 [39, 40]. Figure 5a shows the unrooted tree with the genetic distances on the branches which are fixed in the subsequent relaxed clock analysis in BEAST2.

As an initial starting point, the root is assigned using the midpoint method. After that, according to the genetic distances among seven taxa and the position of the root, consistent divergence times are specified and assigned to each ancestral node, so that a valid rooted time tree is obtained. Once divergence times are determined, rates on the branches are also calculated so that the products match the unrooted substitution tree.

Then we used Constant Distance operator to sample a Markov chain initiated by this starting tree. The resulting posterior distribution is shown in Fig. 5b-d. As can be seen, despite that there is some uncertainty in the root position, the most probable tree in Fig. 5b is consistent with previous analyses of this data (see Figure 2 in Ref. [33]). For large data sets of long sequences, the proposed operators may prove useful to provide faster divergence time estimates based on the assumption of known unrooted topology and branch lengths in units of expected substitutions per site.

## Discussion

We have demonstrated that the presented operator is valid and able to improve the efficiency of phylogenetic MCMC for relaxed clock models. The overall performance of a Bayesian phylogenetic analysis will be affected by the proportion of MCMC steps that this operator is chosen to make the proposal. In the BEAST2 software, this can be changed by modifying the relative weights operators in the operator schedule. The ideal proportion is non-trivial to determine for an arbitrary data set. In this study, we assigned equal weights on operations to all internal nodes (including the root). How to assign weights to achieve better performance is not studied in this paper, and users may assign different weights in practice. Hence, an optimal method of assigning weights still needs further investigation.

The key idea of the presented operator (to maintain the genetic distances) shows a novel direction for more efficient proposals in Bayesian phylogenetic MCMC. For example, the operations on the internal nodes, in the current study, involve one random internal node, one node time and three branch rates. If two or more nodes are selected, then more associated rates and node times can be sampled in one proposal, which may achieve even better efficiency. Another possible approach is to make small changes to the genetic distances as well. To minimise the number of changes to genetic distances, a two-dimensional random draw will be used to change four parameters (one divergence time and one rate changed directly, the other two rates derived so as to minimise changes to genetic distances). What’s more, it should be pointed out that Small Pulley and Big Pulley can only be applied to reversible continuous-time Markov chain models where unrooted trees can be used in inference, because these operators require the underlying unrooted tree to be unchanged. Future work could elaborate a larger class of operators along these lines.

As data sets have increased in size the impetus to improve efficiency of Bayesian phylogenetic inference algorithms has steadily increased. Besides more effective proposal mechanisms within Metropolis-Hastings MCMC, completely novel approaches to Bayesian phylogenetics have also begun to get some attention. Variational methods are one alternative for approximating Bayesian posterior distributions [43]. These approaches make inference an optimisation problem and take advantage of tractable variational distributions that approximate the posterior distribution, thus decreasing the computational cost by avoiding high-dimensional integrals in MCMC sampling schemes. Recent work has investigated the potential for applying variational methods to phylogenetics [44, 45]. Our improved MCMC methods provide a performance baseline for these new approaches.

## Conclusions

As data sets have increased in size, the need for computational efficiency of Bayesian phylogenetic analyses has also increased. In this paper, we have discussed a new tree proposal that substantially increases the efficiency of Bayesian phylogenetic inference under a popular class of relaxed molecular clock models.

We demonstrate the correctness of this algorithm with a series of tests including a well-calibrated simulation study. Based on both simulated and real data sets, the proposed operator is more efficient than current algorithms implemented in BEAST2 for datasets with long sequences. This is a desirable property because efficiency is most important for larger datasets. The proposed operator is available for use as a package of BEAST2.

## Methods

**Fig. 6:**
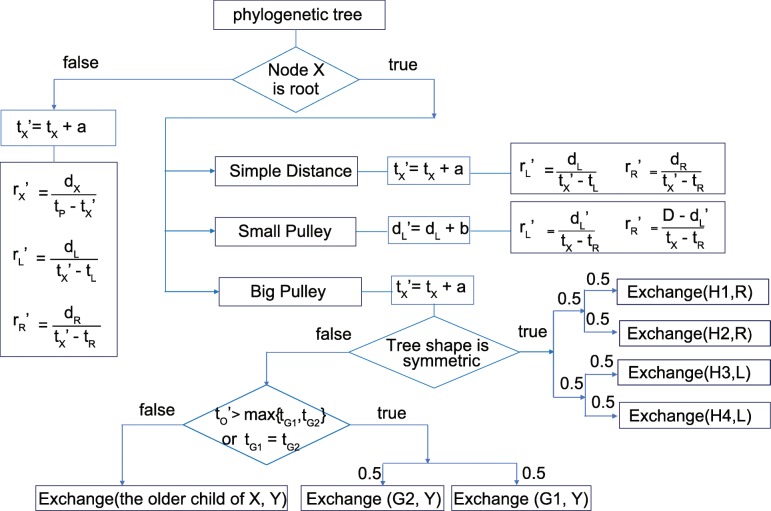
The flow chart of the Constant Distance operator

In this section, we define the Constant Distance Operator. Figure 6 illustrates the flow chart of the proposed operators. In a phylogenetic tree, the node to operate on is denoted by **X** and the Constant Distance Operator works differently on internal nodes and the root node. The details of the operations are introduced step by step in the following subsections.

### Operations on internal nodes

**Fig. 7:**
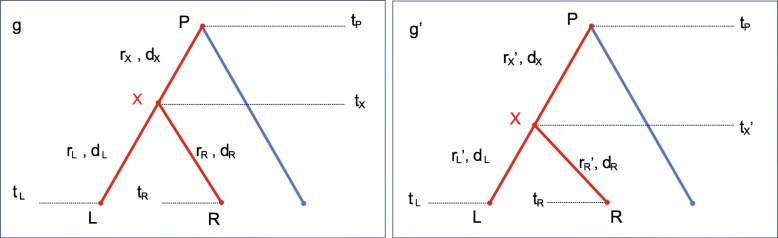
Illustration of the operation on an internal node. The operator proposes *t*_*X*_^′^,*r*_*X*_^′^,*r*_*L*_^′^ and *r*_*R*_^′^, during which *d*_*L*_,*d*_*R*_,*d*_*X*_ are kept constant

Figure 7 represents the tree (or subtree) with the node **X** that is randomly selected from among the internal nodes. Let *g* be the tree in the current state. The following steps propose a new divergence time in *g*^′^ and three rates in **r**^′^.

*Step 1* Identify the parent node and two child nodes of **X**, denoted by **P**, **L** and **R** respectively.

*Step 2* Denote the nodes times of **X**, **P**, **L** and **R** by *t*_*X*_,*t*_*P*_,*t*_*L*_,*t*_*R*_ respectively. Denote the rates on the branches above the nodes by *r*_*X*_,*r*_*L*_ and *r*_*R*_ respectively.

*Step 3* Propose a new node time for **X** by *t*_*X*_^′^←*t*_*X*_+*a*, where *a* follows a Uniform distribution with a symmetric window size *w*, i.e. *a*∼ Uniform[-w, +w], for some window size *w*. Make sure that the proposed time is valid, i.e. max{*t*_*L*_,*t*_*R*_}<*t*_*X*_^′^<*t*_*P*_ holds. Otherwise, we reject the proposal.

*Step 4* Propose new rates by using Eq. 3.

3 $$ \begin{aligned} {r_{X}}' &= \frac{{r_{X}} \times ({{t_{P}} - {t_{X}}})}{{{t_{P}} - {t_{X}}'}} {r_{L}}' = \frac{{{r_{L}} \times ({{t_{X}} - {t_{L}}})}}{{{t_{X}}' - {t_{L}}}}\\ {r_{R}}' &= \frac{{{r_{R}} \times ({{t_{X}} - {t_{R}}})}}{{{t_{X}}' - {t_{R}}}} \end{aligned}  $$

*Step 5* Return the Green ratio *α*_*IN*_ (Refer to *Calculating the Green Ratio* in the following subsection).

### Operations on the root

**Fig. 8:**
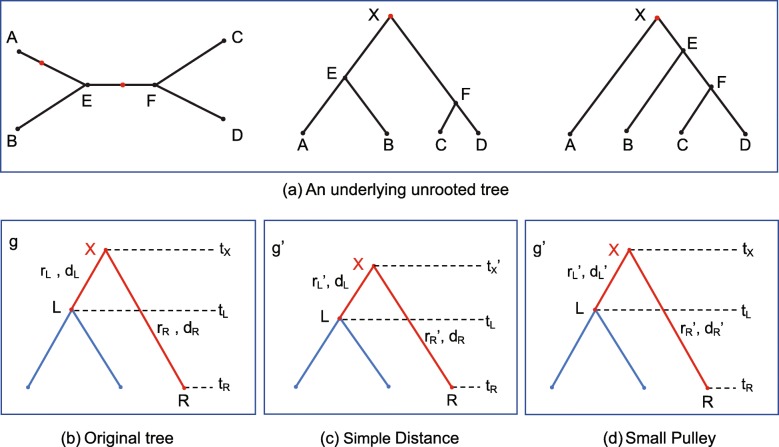
Illustration of operations on root. **a** An example of a 4-taxa unrooted tree and two possible rooted trees for the operator to sample, during which the unrooted tree can not be changed. Based on the original tree in **b**, Simple Distance proposes a node time in *g*^′^ and two rates in **r**^′^ and keeps *d*_*L*_,*d*_*R*_ constant in **c**. Small Pulley proposes two rates in **r**^′^ and *D*=*d*_*L*_+*d*_*R*_ remains constant in **d**

We present three strategies for proposing the new rates and a new divergence time for the special case when **X** is the root node. i) The Simple Distance operator only proposes a new root time. ii) Small Pulley adjusts the distances of branches on both sides of the root. iii) Big Pulley proposes a new tree topology by rearranging the root, without perturbing the unrooted tree. As is illustrated in Fig. 8a, all the operations on the root, including Big Pulley that changes the tree topology, do not change the underlying unrooted tree. For instance, no matter where the root *X* is (either on branch *EF* or *AE*), the operators maintain the distances (*d*_*AB*_,*d*_*AC*_,*d*_*AD*_,*d*_*BC*_,*d*_*BD*_,*d*_*CD*_) and preserve the unrooted tree at the same time.

#### Simple distance

Figure 8b, c and d show the trees that are rooted at the node **X**. The original tree *g* in the current state is shown in Fig. 8b. Similar to the operations on internal nodes, we will use the following steps to propose a new root time in *g*^′^ and two rates in **r**^′^, as is illustrated in Fig. 8c. At the same time, the genetic distances of two branches linked to the root, i.e. *d*_*L*_ and *d*_*R*_, are kept constant.

*Step 1* Identify the child nodes of the root **X**, denoted by **L** and **R**. Their corresponding node times and branch rates are *t*_*X*_,*t*_*L*_,*t*_*R*_ and *r*_*L*_,*r*_*R*_.

*Step 2* Propose a new node time for the root **X** by *t*_*X*_^′^←*t*_*X*_+*a*, where *a*∼ Uniform[-w, +w]. Make sure that *t*_*X*_^′^> max{*t*_*L*_,*t*_*R*_} holds. Otherwise, we reject the proposal.

*Step 3* Propose new rates for branches on both sides of the root by using Eq. 4.

4 $$  {r_{L}}' = \frac{{{r_{L}} \times ({{t_{X}} - {t_{L}}})}}{{{t_{X}}' - {t_{L}}}}\\ {r_{R}}' = \frac{{{r_{R}} \times ({{t_{X}} - {t_{R}}})}}{{{t_{X}}' - {t_{R}}}}  $$

*Step 4* Return the Green ratio *α*_*SD*_.

#### Small pulley

In contrast to Simple Distance, Small Pulley changes genetic distances of branches on both sides of the root. As is illustrated in Fig. 8d, two new rates in **r**^′^ are proposed based on those in the original tree *g*. In order to maintain the total genetic distance *d*_*L*_+*d*_*R*_ of the two branches linked to the root, after *d*_*L*_^′^ is proposed, *d*_*R*_ will be adjusted simultaneously. In other words, Small Pulley keeps *D*=*d*_*L*_+*d*_*R*_ constant. The detailed process includes the following 4 steps.

*Step 1* Identify the child nodes of the root **X**, denoted by **L** and **R**. Their corresponding node times and branch rates are *t*_*X*_,*t*_*L*_,*t*_*R*_ and *r*_*L*_,*r*_*R*_. The implied genetic distances of the two branches linked to the root can be calculated by:

5 $$  {d_{L}} = {r_{L}} \times ({{t_{X}} - {t_{L}}})\\ {d_{R}} = {r_{R}} \times ({{t_{X}} - {t_{R}}})  $$

*Step 2* Propose a new genetic distance for *d*_*L*_ by adding a random number that follows a Uniform distribution, i.e. *d*_*L*_^′^←*d*_*L*_+*b*, where *b*∼ Uniform[-v, +v], for some window size *v*. Make sure that 0<*d*_*L*_^′^<*D* holds. Otherwise, we reject the proposal.

*Step 3* Propose new rates for branches on each side of the root:

6 $$  {r_{L}}' = \frac{{{d_{L}}'}}{{{t_{X}} - {t_{L}}}}\\ {r_{R}}' = \frac{{D - {d_{L}}'}}{{{t_{X}} - {t_{R}}}}  $$

*Step 4* Return the Green ratio *α*_*SP*_.

#### Big pulley

Big Pulley resamples the rates and times while maintaining the implied unrooted tree in units of genetic distance. So the genetic distances between the taxa are held constant, but the location of the root in the time tree is readjusted.

**Fig. 9:**
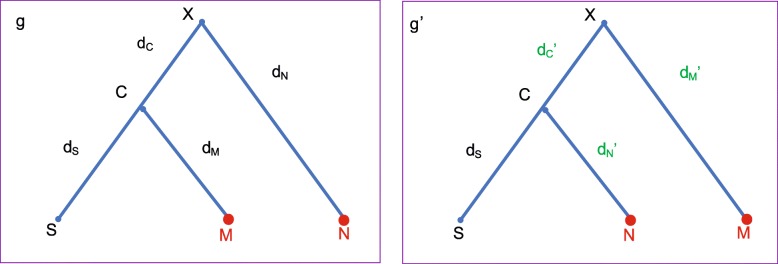
Illustration of *Exchange* (***M***,***N***) method. This method is applied to tree *g* and proposes *g*^′^ by swapping **M** and **N**, so that the three distances are adjusted to maintain the distances among **S**, **M** and **N**. That is, *d*_*C*_^′^=*d*_*C*_+*b,d*_*N*_^′^=*d*_*C*_+*d*_*N*_ and *d*_*M*_^′^=*d*_*M*_−*d*_*C*_^′^, where *b*∼*U*[−*v*,+*v*]
- Propose *d*_*C*_^′^←*d*_*C*_+*b*, where *b*∼ Uniform[-v, +v]. Make sure that 0<*d*_*C*_^′^<*D* holds, where *D*=*d*_*C*_+*d*_*N*_. Otherwise, we reject the proposal.
- The distances on the other three branches, i.e. *d*_*S*_,*d*_*M*_ and *d*_*N*_, will be adjusted:

Before describing the detailed steps, we introduce a method *Exchange* that proposes a new root position. In Fig. 9, let (i) **X** denote the root of tree *g*, (ii) **C** and **N** denote the two child nodes of **X**, (iii) **S** and **M** denote the two child nodes of **C**. The *Exchange*(***M***, ***N***) method involves the following steps:

- Swap the two nodes by pruning and regrafting, i.e. cutting **M** (**N**) at its original position and attaching it to the original position of **N** (**M**).

7 $$ {d_{S}}' = {d_{S}}\\ {d_{M}}' = {d_{M}} - {d_{C}}'\\ {d_{N}}' = {d_{N}} + {d_{C}}  $$

As can be seen from the above descriptions, the method *Exchange*(***M***, ***N***) swaps two nodes and adjusts distances (*d*_*S*_,*d*_*M*_,*d*_*N*_ and *d*_*C*_) on the four branches so as to maintain the implied genetic distances among three taxa **S**, **M** and **N**.

**Fig. 10:**
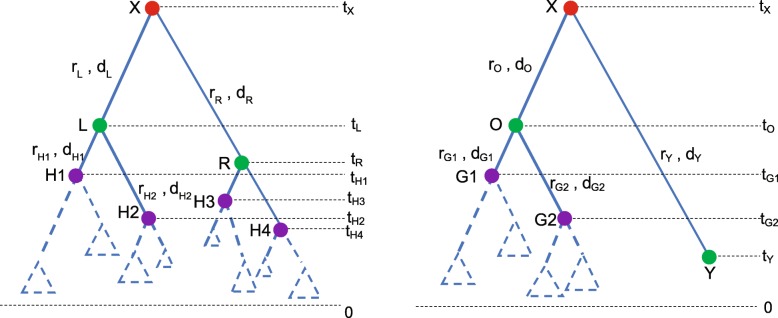
Two different tree shapes. The symmetric tree is on the left and the asymmetric tree is on the right. The dashed triangles represent the potential subtrees rooted at the nodes

Additionally, operations in Big Pulley vary depending on the shape of phylogenetic tree. In Fig. 10, a symmetric tree is shown on the left, in which both the child nodes of the root have two child nodes, i.e. **L** having children **H1**, **H2** and **R** having children **H3**, **H4**. But in the asymmetric tree on the right, only one of the child nodes of the root has two child nodes below it, i.e. **O** having children **G1**, **G2**. But the other child node **Y** doesn’t have any child node, which is a heterochronous tip. The corresponding operations are detailed in the following two parts.

##### Symmetric tree

**Fig. 11:**
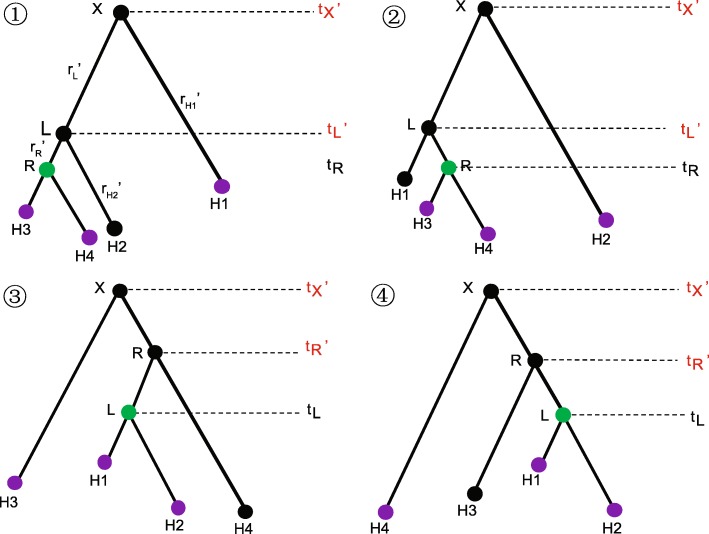
Illustration of operations on the symmetric tree in Fig. 10. The proposed operator will propose one of the four possible trees, each with 0.25 probability

For the symmetric tree in Fig. 10, the operations are illustrated in Fig. 11, after which one of the four possible trees (① ② ③ ④) will be proposed. The detailed process is described in Algorithm 1.

For example, suppose we are going to propose tree ①. After the new node times for the root **X** and **L** are proposed, we apply the method by *Exchange* (***H1***, ***R***), so that four distances are adjusted, as follows:

8 $$ {d_{H1}}' = {d_{H1}} - {d_{L}}' \\ {d_{H2}}' = {d_{H2}} \\ {d_{L}}' = {d_{L}} + b \\ {d_{R}}' = {d_{L}} + {d_{R}}  $$

Finally, in this example the new rates would be updated by:

9 $$ \begin{aligned} {r_{H1}}' = \frac{{{d_{H1}}'}}{{{t_{X}}' - {t_{H1}}}} {r_{H2}}' = \frac{{{d_{H2}}'}}{{{t_{L}}' - {t_{H2}}}} {r_{L}}' = \frac{{{d_{L}}'}}{{{t_{X}}' - {t_{L}}'}} {r_{R}}' = \frac{{{d_{R}}'}}{{{t_{L}}' - {t_{R}}}} \end{aligned}  $$

##### Asymmetric tree

**Fig. 12:**
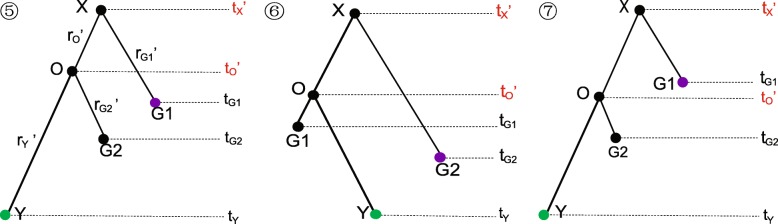
Illustration of operations on the asymmetric tree in Fig. 10. The proposed operator will propose one of the three possible trees. If *t*_*o*_^′^<*t*_*G*1_, ⑦ has 1 probability, otherwise ⑤ and ⑥ have 0.5 probability each


For an asymmetric tree such as in Fig. 10 we would operate as illustrated in Fig. 12, in which there are three possible trees (⑤ ⑥ ⑦). The operations are detailed in Algorithm 2.

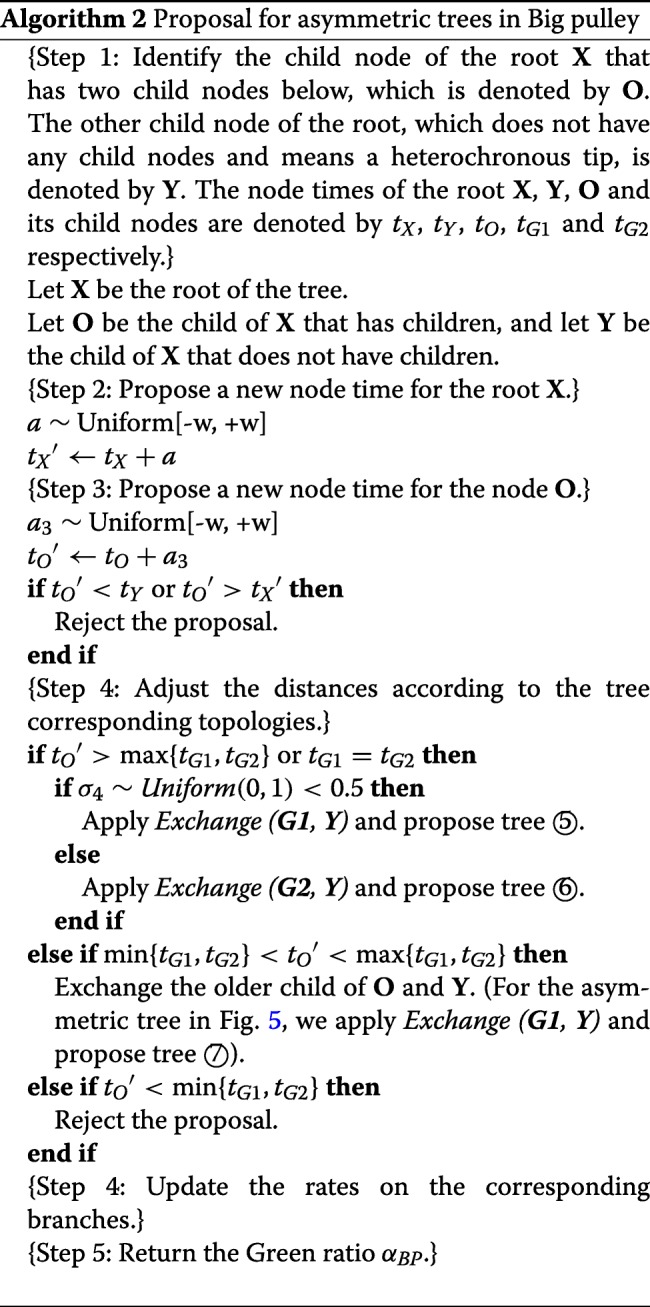


To give an example, assume we are going to propose tree ⑤. Firstly, *t*_*X*_^′^ and *t*_*O*_^′^ are proposed in *Step 3* and *Step 4*. Then, in *Step 4*, the method *Exchange* (***G1***, ***Y***) is applied, after which the four distances are adjusted as follows:

10 $$ {d_{G1}}' = {d_{G1}} - {d_{O}}' \\ {d_{G2}}' = {d_{G2}} \\ {d_{O}}' = {d_{O}} + b \\ {d_{Y}}' = {d_{Y}} + {d_{O}}  $$

And the four rates are updated as follows:

11 $$ \begin{aligned} {r_{G1}}' = \frac{{{d_{G1}}'}}{{{t_{X}}' - {t_{G1}}}} {r_{G2}}' = \frac{{{d_{G2}}'}}{{{t_{O}}' - {t_{G2}}}} {r_{O}}' = \frac{{{d_{O}}'}}{{{t_{X}}' - {t_{O}}'}} {r_{Y}}' = \frac{{{d_{Y}}'}}{{{t_{O}}' - {t_{Y}}}} \end{aligned}  $$

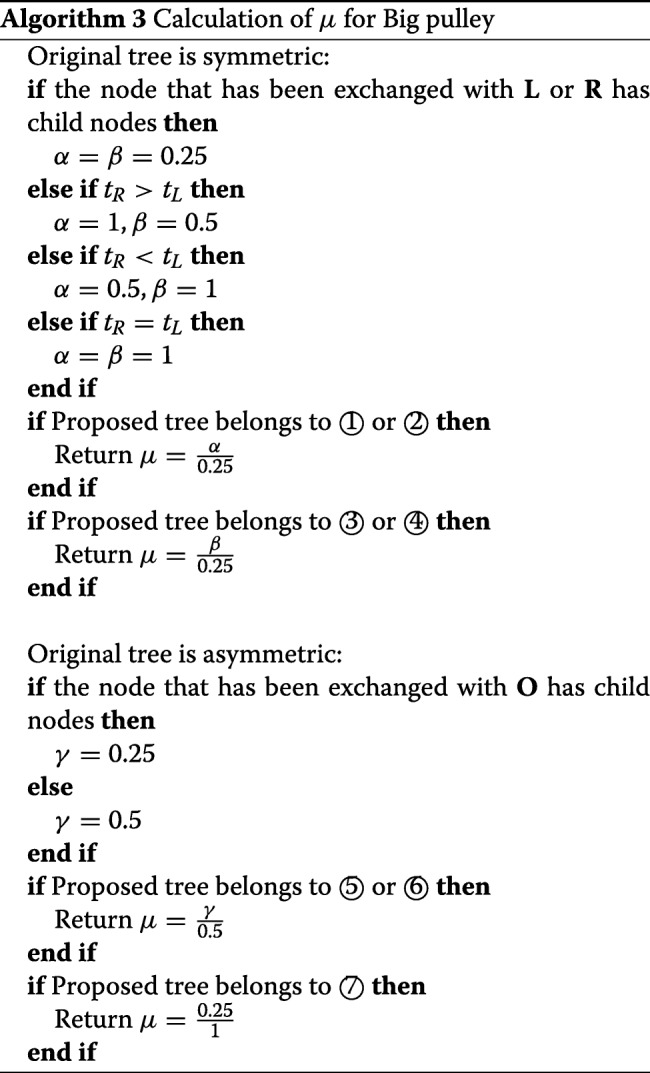


### Calculating the green ratio

MCMC operators must use reversible proposal distributions to satisfy the detailed balance requirements of the MCMC algorithm (Refer to Appendix section 1 for more details). Therefore, all four of our operators involve a final step of calculating the Green ratio for the proposal.

According to the third and fourth steps in the operations for internal nodes, three rates on the branches linked to the selected internal node are proposed by one random number *a* that is used to change the node time. There are four parameters involved in this proposal, comprised of a 3-dimensional rate space and a 1-dimensional time space. The proposed operator utilises one random number in time space and makes changes in both time and rate space, which leads to a dimension-matching problem. To solve this dimension-matching problem, as is mentioned in Green’s paper [20], it is necessary to construct the Jacobian matrix. In Eq. (12), *J*_1_ deals with the parametric spaces before the proposal in vector **IN**=[*t*_*X*_,*r*_*X*_,*r*_*L*_,*r*_*R*_] and after the proposal in vector **OUT**=[*t*_*X*_^′^,*r*_*X*_^′^,*r*_*L*_^′^,*r*_*R*_^′^].

12 $$ {\mathbf{J_{1}}} = \left[ {\begin{array}{cccc} {\frac{{\partial {\mathbf{f}}}}{{\partial {t_{X}}}}}&{\frac{{\partial {\mathbf{f}}}}{{\partial {r_{X}}}}}&{\frac{{\partial {\mathbf{f}}}}{{\partial {r_{L}}}}}&{\frac{{\partial {\mathbf{f}}}}{{\partial {r_{R}}}}} \end{array}} \right] = \left[ {\begin{array}{cccc} {\frac{{\partial {f_{1}}}}{{\partial {t_{X}}}}}&{\frac{{\partial {f_{1}}}}{{\partial {r_{X}}}}}&{\frac{{\partial {f_{1}}}}{{\partial {r_{L}}}}}&{\frac{{\partial {f_{1}}}}{{\partial {r_{R}}}}} \\ {\frac{{\partial {f_{2}}}}{{\partial {t_{X}}}}}&{\frac{{\partial {f_{2}}}}{{\partial {r_{X}}}}}&{\frac{{\partial {f_{2}}}}{{\partial {r_{L}}}}}&{\frac{{\partial {f_{2}}}}{{\partial {r_{R}}}}} \\ {\frac{{\partial {f_{3}}}}{{\partial {t_{X}}}}}&{\frac{{\partial {f_{3}}}}{{\partial {r_{X}}}}}&{\frac{{\partial {f_{3}}}}{{\partial {r_{L}}}}}&{\frac{{\partial {f_{3}}}}{{\partial {r_{R}}}}} \\ {\frac{{\partial {f_{4}}}}{{\partial {t_{X}}}}}&{\frac{{\partial {f_{4}}}}{{\partial {r_{X}}}}}&{\frac{{\partial {f_{4}}}}{{\partial {r_{L}}}}}&{\frac{{\partial {f_{4}}}}{{\partial {r_{R}}}}} \end{array}} \right]\text{,}  $$

where the functions *f*_1_,*f*_2_,*f*_3_ and *f*_4_ represent how the operator makes a proposal. After substituting Eq. (3) in Eq. (12), the Green ratio for the internal nodes can be derived:

13 $$ {}{\alpha_{IN}} = \frac{{p (- a)}}{{p (a)}}\left| {\mathbf{J_{1}}} \right| = \frac{{{t_{P}} - {t_{X}}}}{{{t_{P}} - {t_{X}}'}} \times \frac{{{t_{X}} - {t_{L}}}}{{{t_{X}}' - {t_{L}}}} \times \frac{{{t_{X}} - {t_{R}}}}{{{t_{X}}' - {t_{R}}}}\text{,}  $$

where the proposal density *p*(−*a*) is equal to *p*(*a*) since the random number *a* is drawn from Uniform distribution.

Likewise, the Green ratio for Simple Distance, Small Pulley and Big Pulley can be obtained:

14 $$ {\alpha_{SD}} = \frac{{{t_{X}} - {t_{L}}}}{{{t_{X}}' - {t_{L}}}} \times \frac{{{t_{X}} - {t_{R}}}}{{{t_{X}}' - {t_{R}}}}\text{,}  $$

15 $$ {\alpha_{SP}} = 1\text{,}  $$

16 $$ {}{\alpha_{BP}} = \mu \times \frac{{{t_{X}}' - {t_{C}}}}{{{t_{X}}' - {t_{C}}'}} \times \frac{{{t_{C}} - {t_{S}}}}{{{t_{C}}' - {t_{S}}}} \times \frac{{{t_{C}} - {t_{N1}}}}{{{t_{X}}' - {t_{N1}}}} \times \frac{{{t_{X}} - {t_{N2}}}}{{{t_{C}}' - {t_{N2}}}}\text{,}  $$

where *μ*=*p*(*g*^′^,*g*)/*p*(*g,g*^′^) is defined as the proposal ratio of topology change and is obtained by Algorithm 3. More details of how to calculate the determinant of the Jacobian matrix are explained in Appendix1 section.

## Appendix

### 1. the green ratio

When developing an operator for MCMC, the proposal function must be reversible. In other words, the probability that the operator propose a new state from the current state is required to be equal to the probability that the proposed state goes back to current state. To be specific, let *π*(*x*) be the target probability distribution and *p*(*x*,*x*^′^) be the transition kernel in the continuous Markov chain. The reversibility condition requires that *π*(*x*)*p*(*x*,*x*^′^)=*π*(*x*^′^)*p*(*x*^′^,*x*). And an operator provides a proposal *q*(*x*,*x*^′^) with some probability *α*(*x*,*x*^′^) that the proposal is accepted. Thus, the reversibility condition is rewritten as *π*(*x*)*q*(*x*,*x*^′^)*α*(*x*,*x*^′^)=*π*(*x*^′^)*q*(*x*^′^,*x*)*α*(*x*^′^,*x*).

Considering the subspace *φ*_1_ on *x* and subspace *φ*_2_ on *x*^′^, it is assumed that there is a symmetric measure on the combined parametric space *φ*=*φ*_1_×*φ*_2_, so that *π*(*x*)*q*(*x*,*x*^′^) has a density with respect to a single measure on *φ*. Then, Green suggested that the reversibility condition should be satisfied by detailed balance [20], as represented by Eq. (17). And according to Peskun’ proof, it is optimal to take Eq. (18) as the acceptance probability to retain the detailed balance [46].

17 $$ \begin{aligned} \int_{A} {\pi (x) d_{x}} {\int_{B} {q(x, x')}{\alpha(x, x')} d_{x}} = \int_{B} {\pi (x') d_{x'}}{\int_{A} {q(x', x)}{\alpha(x', x)} d_{x'}} \text{,} \end{aligned}  $$

where *A*∈*φ*_1_ and *B*∈*φ*_2_ are two Borel sets. *q*(*x*,*x*^′^) denotes the probability that the operator proposes a new state *x*^′^ given the current state *x*.

18 $$ {\alpha_{H}}(x, x') = \min \left\{ {1,\frac{{\pi (x'){p}(x',x)}}{{\pi (x){p}(x,x')}}} \right\} \text{,}  $$

where *p*(*x*^′^,*dx*)/*p*(*x*,*dx*^′^) is known as the Hastings ratio.

However, for operators that do not have a symmetric measure, it is necessary to include the Jacobian matrix **J** in order to deal with the dimension matching problem, as is discussed in Green’s paper [20]. In this case, Eq. (18) is extended, as is shown in Eq. (19).

19 $$ {\alpha_{G}}(x, x') = \min \left\{ {1,\frac{{\pi (x'){p}(x',x)}}{{\pi (x){p}(x,x')}}}\left|{\mathbf{J}}\right| \right\} \text{,}  $$

where **J**=∇*h*(*x*,*x*^′^) represents a vector differential matrix of deterministic function *h*. $\alpha = \frac {{p}(x',x)}{{p}(x,x')}\left |{\mathbf {J}}\right |$ is defined as the Green ratio, and **J** ensures that the proposal have a symmetric measure on each subspace in state *x* and *x*^′^.

### 1.1 calculating the green ratio for operations on internal nodes

The Constant Distance Operator firstly proposes a new time for the randomly selected internal node (Eq. (20a)), and then proposes three rates by the original distances and new node times(Eqs. (20b) ∼ (20d)).

20a $$ {f_{1}}:{{\mathrm{t}}_{X}}{\text{' = }}{{\mathrm{t}}_{X}}{\text{ + a}}  $$

20b $$ {f_{2}}:{r_{X}}' = \frac{{{r_{X}} \times ({t_{P}} - {t_{X}})}}{{{t_{P}} - {t_{X}}'}}  $$

20c $$ {f_{3}}:{r_{L}}' = \frac{{{r_{L}} \times ({t_{X}} - {t_{L}})}}{{{t_{X}}' - {t_{L}}}}  $$

20d $$ {f_{4}}:{r_{R}}' = \frac{{{r_{R}} \times ({t_{X}} - {t_{2}})}}{{{t_{X}}' - {t_{R}}}}  $$

Substituting Eq. (20) in the Jacobian matrix **J**_1_ (Eq. (12)), we can get Eq. (21), so that the determinant of **J**_1_ can be obtained by Eq. (22).

21 $$ {{\mathbf{J}}_{1}} = \left[ {\begin{array}{cccc} 1&0&0&0 \\ {\frac{{ - {r_{X}}}}{{{t_{P}} - {t_{X}}'}}}&{\frac{{{t_{P}} - {t_{X}}}}{{{t_{P}} - {t_{X}}'}}}&0&0 \\ {\frac{{{r_{L}}}}{{{t_{X}}' - {t_{L}}}}}&0&{\frac{{{t_{X}} - {t_{L}}}}{{{t_{X}}' - {t_{L}}}}}&0 \\ {\frac{{{r_{R}}}}{{{t_{X}}' - {t_{R}}}}}&0&0&{\frac{{{t_{X}} - {t_{R}}}}{{{t_{X}}' - {t_{R}}}}} \end{array}} \right]  $$

22 $$ \begin{aligned} \left| {{{\mathbf{J}}_{1}}} \right| &= 1 \times \left| {\begin{array}{ccc} {\frac{{{t_{P}} - {t_{X}}}}{{{t_{P}} - {t_{X}}'}}}&0&0 \\ 0&{\frac{{{t_{X}} - {t_{L}}}}{{{t_{X}}' - {t_{L}}}}}&0 \\ 0&0&{\frac{{{t_{X}} - {t_{R}}}}{{{t_{X}}' - {t_{R}}}}} \end{array}} \right| \\&= \frac{{{t_{P}} - {t_{X}}}}{{{t_{P}} - {t_{X}}'}} \times \left| {\begin{array}{cc} {\frac{{{t_{X}} - {t_{L}}}}{{{t_{X}}' - {t_{L}}}}}&0 \\ 0&{\frac{{{t_{X}} - {t_{R}}}}{{{t_{X}}' - {t_{R}}}}} \end{array}} \right| \\&= \frac{{{t_{P}} - {t_{X}}}}{{{t_{P}} - {t_{X}}'}} \times \frac{{{t_{X}} - {t_{L}}}}{{{t_{X}}' - {t_{L}}}} \times \frac{{{t_{X}} - {t_{R}}}}{{{t_{X}}' - {t_{R}}}} \end{aligned}  $$

### 1.2 calculating the green ratio for simple distance

Simple Distance proposes two rates by using Eqs. (23b) and (23c), according the new root time in Eq. (23a). So the Jacobian matrix can be obtained as is shown in Eq. (24).

23a $$ {t_{X}}' = {t_{X}} + a  $$

23b $$  {r_{L}}' = \frac{{{r_{L}} \times ({t_{X}} - {t_{L}})}}{{{t_{X}}' - {t_{L}}}}  $$

23c $$ {r_{R}}' = \frac{{{r_{R}} \times ({t_{X}} - {t_{R}})}}{{{t_{X}}' - {t_{R}}}}  $$

24 $$ {{\mathbf{J}}_{2}} = \left[ {\begin{array}{ccc} {\frac{{\partial {t_{X}}'}}{{\partial {t_{X}}}}}&{\frac{{\partial {t_{X}}'}}{{\partial {r_{X}}}}}&{\frac{{\partial {t_{X}}'}}{{\partial {r_{R}}}}} \\ {\frac{{\partial {r_{L}}'}}{{\partial {t_{X}}}}}&{\frac{{\partial {r_{L}}'}}{{\partial {r_{X}}}}}&{\frac{{\partial {r_{L}}'}}{{\partial {r_{R}}}}} \\ {\frac{{\partial {r_{X}}'}}{{\partial {t_{X}}}}}&{\frac{{\partial {r_{X}}'}}{{\partial {r_{X}}}}}&{\frac{{\partial {r_{X}}'}}{{\partial {r_{R}}}}} \end{array}} \right] = \left[ {\begin{array}{ccc} 1&0&0 \\ {\frac{{{r_{L}}}}{{{t_{X}}' - {t_{L}}}}}&{\frac{{{t_{X}} - {t_{L}}}}{{{t_{X}}' - {t_{L}}}}}&0 \\ {\frac{{{r_{x}}}}{{{t_{X}}' - {t_{R}}}}}&0&{\frac{{{t_{X}} - {t_{R}}}}{{{t_{X}}' - {t_{R}}}}} \end{array}} \right]  $$

So the determinant of **J**_2_ is calculated by Eq. (25)

25 $$ \left| {{{\mathbf{J}}_{2}}} \right| = \frac{{{t_{X}} - {t_{L}}}}{{{t_{X}}' - {t_{L}}}} \times \frac{{{t_{X}} - {t_{R}}}}{{{t_{X}}' - {t_{R}}}}  $$

### Calculating the green ratio for small pulley

Small Pulley proposes a new genetic distance of a branch on one side of the root by adding a random number *b*, which is equal to adding a random number *b* to the original product of rate and time on that branch. As a result, a new rate is proposed by Eq. (26a). Similarly, a new rate on another branch is proposed by Eq. (26b), because the total distance of the two branches linked to the root should remain constant.

26a $$ {r_{L}}' = \frac{{{r_{L}} \times ({t_{X}} - {t_{L}}) + b}}{{{t_{X}} - {t_{L}}}}  $$

26b $$ \begin{aligned} {r_{R}}' &= \frac{{[{r_{R}} \times ({t_{X}} - {t_{R}}) + {r_{L}} \times ({t_{X}} - {t_{L}})] - [{r_{L}} \times ({t_{X}} - {t_{L}}) + b]}}{{{t_{X}} - {t_{R}}}}\\& = \frac{{{r_{R}} \times ({t_{X}} - {t_{R}}) - b}}{{{t_{X}} - {t_{R}}}} \end{aligned}  $$

Then, as is illustrated in Eq. (27), the Jacobian matrix **J**_3_ is simply obtained, which makes the determinant |**J**_3_|=1.

27 $$ {{\mathbf{J}}_{3}} = \left[ {\begin{array}{cc} {\frac{{\partial {r_{L}}'}}{{\partial {r_{L}}}}}&{\frac{{\partial {r_{L}}'}}{{\partial {r_{X}}}}} \\ {\frac{{\partial {r_{R}}'}}{{\partial {r_{L}}}}}&{\frac{{\partial {r_{X}}'}}{{\partial {r_{X}}}}} \end{array}} \right] = \left[ {\begin{array}{cc} 1&0 \\ 0&1 \end{array}} \right]  $$

### 1.3 calculating the green ratio for big pulley

Two new node times are proposed in Big Pulley. One is the root time (Eq. (28a)), the other is the node time of the child node of the root. It can be either children of the root, i.e. **son** and **dau**. So *t*_*C*_^′^ is used to denote the node time proposed, as is seen in Eq. (28b). In addition, the distances are adjusted by the method *Exchange* (***M***, ***N***), dependent on which nodes are chosen. As a result, the four rates are proposed, as is shown in Eq. (28c) ∼Eq. (28f)

28a $$ {t_{X}}' = {t_{X}} + a  $$

28b $$ {t_{C}}' = {t_{C}} + {a_{1,2,3}}  $$

28c $$ {r_{C}}' = \frac{{{r_{C}} \times (t{}_{X} - {t_{C}}) + b}}{{t{}_{X}' - {t_{C}}'}}  $$

28d $$ {r_{S}}' = \frac{{{r_{2}} \times (t{}_{C} - {t_{S}})}}{{t{}_{C}' - {t_{S}}}}  $$

28e $$ {r_{M}}' = \frac{{{r_{M}} \times ({t_{C}} - {t_{M}}) - [{r_{C}} \times ({t_{X}} - {t_{C}}) + b]}}{{{t_{X}}' - {t_{M}}}}  $$

28f $$ {r_{N}}' = \frac{{{r_{C}} \times ({t_{X}} - {t_{C}}) + {r_{N}} \times ({t_{X}} - {t_{N}})}}{{{t_{C}}' - {t_{N}}}}  $$

where *a*_1,2,3_ is the random number to propose a new node time for the child node of the root. Depending on which child node is selected, the notation is different, i.e. *a*_1_,*a*_2_,*a*_3_. Here, to make it a general case, *a*_*x*_ is used.

**Fig. 13:**
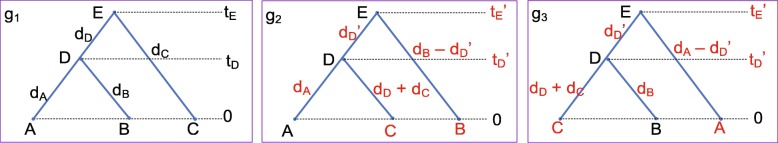
The illustration of sampling from prior. *g*_1_ is set to be the original tree where an MCMC chain starts. When testing Big Pulley, the proposed operator samples the trees among *g*_1_,*g*_2_ and *g*_3_

Therefore, the Jacobian matrix **J**_4_ for the six parameters in Eq. (28) is obtained by Eq. (29). And the determinant of **J**_4_ is calculated shown in Eq. (30).

29 $$ {}\begin{aligned} {{\mathbf{J}}_{4}} &= \left[ {\begin{array}{cccccc} {\frac{{\partial {t_{X}}'}}{{\partial {t_{X}}}}}&{\frac{{\partial {t_{X}}'}}{{\partial {t_{C}}}}}&{\frac{{\partial {t_{X}}'}}{{\partial {r_{C}}}}}&{\frac{{\partial {t_{X}}'}}{{\partial {r_{S}}}}}&{\frac{{\partial {t_{X}}'}}{{\partial {r_{M}}}}}&{\frac{{\partial {t_{X}}'}}{{\partial {r_{N2}}}}} \\ {\frac{{\partial {t_{C}}'}}{{\partial {t_{X}}}}}&{\frac{{\partial {t_{C}}'}}{{\partial {t_{C}}}}}&{\frac{{\partial {t_{C}}'}}{{\partial {r_{C}}}}}&{\frac{{\partial {t_{C}}'}}{{\partial {r_{S}}}}}&{\frac{{\partial {t_{C}}'}}{{\partial {r_{M}}}}}&{\frac{{\partial {t_{C}}'}}{{\partial {r_{N2}}}}} \\ {\frac{{\partial {r_{C}}'}}{{\partial {t_{X}}}}}&{\frac{{\partial {r_{C}}'}}{{\partial {t_{C}}}}}&{\frac{{\partial {r_{C}}'}}{{\partial {r_{C}}}}}&{\frac{{\partial {r_{C}}'}}{{\partial {r_{S}}}}}&{\frac{{\partial {r_{C}}'}}{{\partial {r_{M}}}}}&{\frac{{\partial {r_{C}}'}}{{\partial {r_{N2}}}}} \\ {\frac{{\partial {r_{S}}'}}{{\partial {t_{X}}}}}&{\frac{{\partial {r_{S}}'}}{{\partial {t_{C}}}}}&{\frac{{\partial {r_{S}}'}}{{\partial {r_{C}}}}}&{\frac{{\partial {r_{S}}'}}{{\partial {r_{S}}}}}&{\frac{{\partial {r_{S}}'}}{{\partial {r_{M}}}}}&{\frac{{\partial {r_{S}}'}}{{\partial {r_{N2}}}}} \\ {\frac{{\partial {r_{M}}'}}{{\partial {t_{X}}}}}&{\frac{{\partial {r_{M}}'}}{{\partial {t_{C}}}}}&{\frac{{\partial {r_{M}}'}}{{\partial {r_{C}}}}}&{\frac{{\partial {r_{M}}'}}{{\partial {r_{S}}}}}&{\frac{{\partial {r_{M}}'}}{{\partial {r_{M}}}}}&{\frac{{\partial {r_{M}}'}}{{\partial {r_{N}}}}} \\ {\frac{{\partial {t_{N}}'}}{{\partial {t_{X}}}}}&{\frac{{\partial {t_{N}}'}}{{\partial {t_{C}}}}}&{\frac{{\partial {t_{N}}'}}{{\partial {r_{C}}}}}&{\frac{{\partial {t_{N}}'}}{{\partial {r_{S}}}}}&{\frac{{\partial {t_{N}}'}}{{\partial {r_{N}}}}}&{\frac{{\partial {t_{N}}'}}{{\partial {r_{N}}}}} \end{array}} \right] \\&= \left[ {\begin{array}{cccccc} 1&0&0&0&0&0 \\ 0&1&0&0&0&0 \\ {\frac{{{r_{C}}}}{{{t_{X}}' - {t_{C}}'}}}&{\frac{{ - {r_{C}}}}{{{t_{X}}' - {t_{C}}'}}}&{\frac{{{t_{X}}' - {t_{C}}}}{{{t_{X}}' - {t_{C}}'}}}&0&0&0 \\ 0&{\frac{{{r_{S}}}}{{t' - {t_{S}}}}}&0&{\frac{{{t_{C}} - {t_{S}}}}{{{t_{C}}' - {t_{S}}}}}&0&0 \\ {\frac{{ - {r_{C}}}}{{{t_{X}}' - {t_{M}}}}}&{\frac{{{r_{N1}} + {r_{C}}}}{{{t_{X}}' - {t_{M}}}}}&{\frac{{ - ({t_{X}} - {t_{C}})}}{{{t_{X}}' - {t_{M}}}}}&0&{\frac{{{t_{C}} - {t_{M}}}}{{{t_{X}}' - {t_{M}}}}}&0 \\ {\frac{{{r_{C}} + {r_{S}}}}{{{t_{C}}' - {t_{N}}}}}&{\frac{{ - ({r_{C}} + {r_{S}})}}{{{t_{C}}' - {t_{N}}}}}&{\frac{{{t_{X}} - {t_{C}}}}{{{t_{C}}' - {t_{N}}}}}&0&0&{\frac{{{t_{X}} - {t_{N}}}}{{{t_{C}}' - {t_{N}}}}} \end{array}} \right] \end{aligned}  $$

30 $$ \left| {{{\mathbf{J}}_{4}}} \right| = \frac{{{t_{X}}' - {t_{C}}}}{{{t_{X}}' - {t_{C}}'}} \times \frac{{{t_{C}} - {t_{S}}}}{{{t_{C}}' - {t_{S}}}} \times \frac{{{t_{C}} - {t_{M}}}}{{{t_{X}}' - {t_{M}}}} \times \frac{{{t_{X}} - {t_{N}}}}{{{t_{C}}' - {t_{N}}}}  $$

Last but not least, due to the change of tree topology in *Exchange* (***M***, ***N***), the probability of the proposed tree going back to the original tree *p*(*g*|*g*^′^), as well as the probability of making the proposal *p*(*g*^′^|*g*), should be considered. As the ratio of *p*(*g*|*g*^′^)/*p*(*g*^′^|*g*) is defined as *μ*, the calculation of *μ* is detailed in the following algorithm.

### 2. sampling from the prior

In this section, we aim to validate the correctness of the proposed operators. To be more specific, we firstly run the simulations by sampling from prior distributions in BEAST2. Since the prior distributions are deterministic, we can analytically calculate the theoretical joint-distributions of sampled parameters in MCMC chains. By comparing the sampled distributions with the analytical results, we demonstrate whether the proposed operators are able to sample parameters correctly.

In Fig. 13, a tree with three taxa *A*, *B* and *C* (plus one internal node *D*, and root *E*) is used as a small example in the experiments in this section. In the figure, *g*_1_ is set as the initial tree. Firstly, a LogNormal distribution is used as the rate prior in the uncorrelated relaxed clock model, given by Eq. (31).

31 $$ {}r = \{{r_{A}}\quad{r_{B}}\quad{r_{C}}\quad{r_{D}}\} \sim LogNormal(m = -3, s = 0.25)  $$

In addition, a Coalescent model [47] with constant population size (*N*=0.3) is used to describe the tree prior. Hence, for the tree in Fig. 13, the probability of node times is calculated by Eq. (32).

32 $$ p(t=\{{t_{E}},{t_{D}}\}) = (\frac{1}{N} \times {e^{- \frac{1}{N}({t_{E}} - {t_{D}})}}) \times (\frac{1}{N} \times {e^{- \frac{3}{N}{t_{D}}}})  $$

**Table 3:** Initial settings for testing operations on internal nodes

	Genetic distances (fixed)				*t*_*D*_	*t*_*E*_	Initial rates			
	*d*_*j*_	*d*_*k*_	*d*_*x*_	*d*_*i*_	initial	(fixed)	*r*_*j*_	*r*_*k*_	*r*_*x*_	*r*_*i*_
Scenario 1	0.1	0.2	0.4	0.27	1	10	0.1	0.2	0.04	0.03
Scenario 2	0.4	0.8	2.4	1.6	0.4	0.8	1	2	3	4

**Table 4:** Results of sampling the internal node

	Chain Length	Sample from MCMC			Integral curve			Plot
		Mean	Err	St.dev	Mean	Err	St.dev	
Scenario 1	10000000	3.2727	8.3e-3	0.5467	3.2669	1.3e-06	0.5553	Fig. 14a
	20000000	3.271	6.1e-3	0.5616				Fig. 14b
Scenario 2	10000000	0.4677	3.9e-04	0.0265	0.4667	3.5e-05	0.0262	Fig. 14c
	20000000	0.4672	2.8e-04	0.0262				Fig. 14d

After the priors are specified, the distribution to sample can be exactly known, since the samples are drawn from the prior distributions. In other words, as the rates are functions of its genetic distance and times, the joint distribution to sample can be represented by Eq. (33).

33 $$ \begin{aligned} p(r,t) &= p({t_{E}},{t_{D}}) \times p({r_{D}}) \times p({r_{A}}) \times p({r_{B}}) \times p({r_{C}}) \\&= p({t_{E}},{t_{D}}) \times p\left(\frac{{{d_{D}}}}{{t_{E}} - {t_{D}}}\right) \times p\left(\frac{{{d_{A}}}}{{t_{D}} - {t_{A}}}\right)\\& \quad\times p\left(\frac{{{d_{B}}}}{{t_{D}} - {t_{B}}}\right) \times p\left(\frac{{{d_{C}}}}{{t_{E}} - {t_{C}}}\right)\text{,} \end{aligned}  $$

where *p*(.) is the probability of certain rate values in the LogNormal distribution. Therefore, the whole probability can be obtained by conducting numerical integration on Eq. (33), which shows the probability distribution over all the possible values of parameters.

#### 2.1 test the operator on internal nodes

The genetic distances, node times and rates for *g*_1_ in Fig. 13 are given in Table 3. To test roundly, two scenarios are designed. In each scenario, the genetic distances are fixed, the node time *t*_*D*_ starts from the initial value and will be changed by the proposed operator during the sampling process. Essentially, the proposed operator makes node *D* move between node *A* and *E*. Besides, to make sure that the result is robust, two different MCMC chain lengths are performed in each scenario, i.e. 10 million and 20 million.

**Fig. 14:**
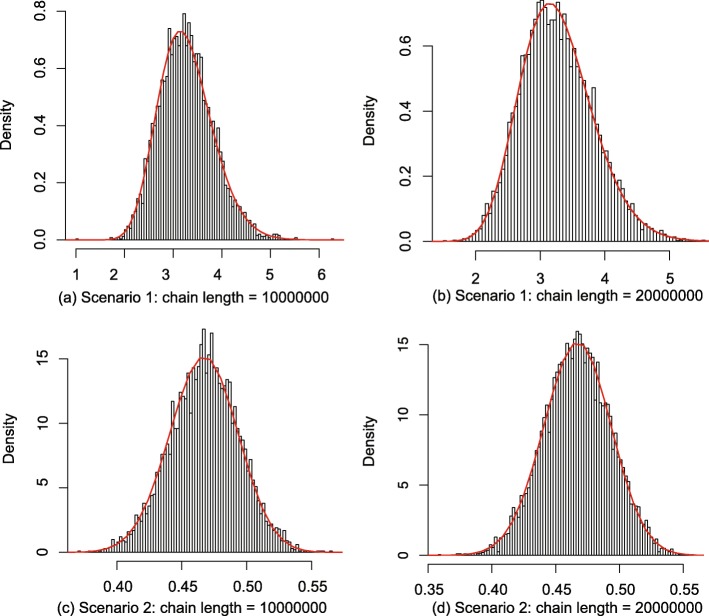
Sampled parameters in tests of internal nodes. The horizontal axis represents the node time of D in Fig. 13. The two scenarios sample two trees with different distances specified in Table ??

The mean, mean error and the standard deviation of the MCMC samples are summarised in Table 4. Besides, according to Eq. (33), the actual joint distribution is obtained by using Eq. (34), and is used to evaluate the results, which is also included in Table 4. Moreover, the histograms of MCMC samples that indicate the sampled distributions, as well as the curves of the numerical integration of Eq. (34), are shown in Fig. 14. From Table 4 and Fig. 14, it can be seen that the red curves well fit the black histograms, and the mean values and standard deviations are consistent, which makes it safe to conclude that the proposed operator samples the internal node correctly.

34 $$ \begin{aligned} p(r,t) &= \int_{{t_{D}} = 0}^{{t_{E}}} {p({t_{E}},{t_{D}})} \times p\left(\frac{d_{A}}{t_{D}}\right) \times p\left(\frac{d_{B}}{t_{D}}\right)\\ & \times p\left(\frac{d_{D}}{{t_{E}} - {t_{D}}}\right) \times p\left(\frac{d_{C}}{t_{E}}\right){d_{t_{D}}} \end{aligned}  $$

#### 2.2 test the operator on root

Still starting from *g*_1_ in Fig. 13, the initial settings for testing the root are given in Table 5. And the three strategies are tested separately in the following parts.

**Table 5:** Initial settings for operations on the root

Strategy	Genetic distances				*t*_*D*_	*t*_*E*_	Initial rates			
	*d*_*j*_	*d*_*k*_	*d*_*x*_	*d*_*i*_			*r*_*j*_	*r*_*k*_	*r*_*x*_	*r*_*i*_
Simple Distance	0.1	0.2	0.4	0.27	1	10	0.1	0.2	0.04	0.03
Small Pulley	0.1	0.2	0.67		1	10	0.1	0.2	0.04	0.03
Big Pulley	0.5	0.5	0.5		5	10	0.1	0.1	0.03	0.04

##### 2.2.1 Using Simple Distance

The root time *t*_*E*_ is sampled by Simple Distance, which ranges from 1 to positive infinity theoretically. Namely, all the genetic distances and the node time *t*_*D*_ are fixed. Similar to Eq. (34), the joint distribution of *t*_*E*_ and rates to sample can be obtained by Eq. (35).

35 $$ {}\begin{aligned} p(r,t) &= \int_{{t_{E}} = 1}^{+ \infty} {p({t_{E}},{t_{D}})} \times p\left(\frac{{{d_{A}}}}{{{t_{D}}}}\right) \times p\left(\frac{{{d_{B}}}}{{{t_{D}}}}\right)\\ & \times p\left(\frac{{{d_{D}}}}{{{t_{E}} - {t_{D}}}}\right) \times p\left(\frac{{{d_{C}}}}{{{t_{E}}}}\right){d_{t_{E}}} \end{aligned}  $$

**Fig. 15:**
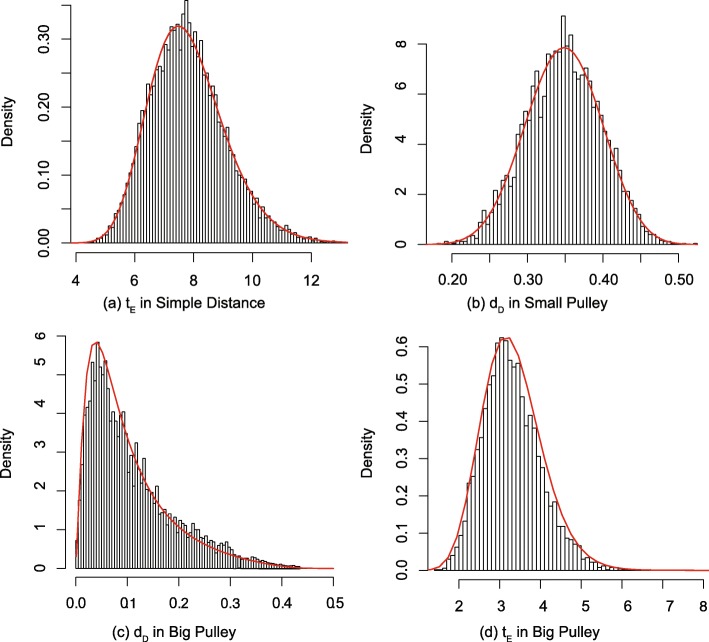
Sampled parameters in test of the root. For the trees in Fig. 13, Simple Distance samples the root time *t*_*E*_ only, Small Pulley samples the distance *d*_*D*_ only, and Big Pulley samples *t*_*E*_,*t*_*D*_,*d*_*D*_. To make it simple, *t*_*E*_ and *d*_*D*_ are compared

The results are given in Table 6 and Fig. 15a. As can be seen, the mean and the standard deviation of MCMC samples and numerical integration are close to each other, which confirms that the two distribution are the same. Thus, Simple Distance samples the root time and two branch rates correctly.

**Table 6:** Results of sampling the root

Strategy	Variable	Sample from MCMC		Integral curve		Plot
		Mean	St.dev	Mean	St.dev	
Simple Distance	*t*_*E*_	7.8081	1.2884	7.8187	1.2992	Fig. 15a
Small Pulley	*d*_*i*_	0.3480	0.0492	0.3476	0.0494	Fig. 15b
Big Pulley	*d*_*i*_	0.1016	0.0766	0.0960	0.0760	Fig. 15c
	*t*_*E*_	3.3017	0.6908	3.3095	0.6912	Fig. 15d

##### 2.2.2 Using Small Pulley

Although both *d*_*x*_ and *d*_*i*_ are changed during the sampling process when using Small Pulley, the sum of *d*_*D*_ and *d*_*C*_ are kept 0.67 in this test, as the initial setting shown in Table 5. To make it simple, only *d*_*D*_ is compared.

Then, based on Eq. (33), the exact distribution of *d*_*i*_ can be obtained by Eq. (36), which is compared with the sampled distribution in Table 6 and Fig. 15b. Even though there exist some errors, the sampled parameters can be considered to follow the same distribution. So the Small Pulley is also able to provide correct samples.

36 $$ \begin{aligned} p(r,t) &= \int_{{d_{D}} = 1}^{0.67} {p({t_{E}},{t_{D}})} \times p\left(\frac{{{d_{A}}}}{{{t_{D}}}}\right) \times p\left(\frac{{{d_{B}}}}{{{t_{D}}}}\right) \times p\left(\frac{{{d_{D}}}}{{{t_{E}} - {t_{D}}}}\right)\\ & \times p\left(\frac{{0.67 - {d_{D}}}}{{{t_{E}}}}\right){d_{d_{D}}} \end{aligned}  $$

##### 2.2.3 Using Big Pulley

For *g*_1_ in Fig. 13, a new tree, together with the root time *t*_*E*_ and node time of its older child *t*_*D*_, as well as a genetic distance *d*_*i*_, is proposed by Big Pulley. In this case, the initial tree *g*_1_ will either go to *g*_2_ or *g*_3_, as is shown in Fig. 13. So the samples are repeatedly drawn from the 3 trees. Besides, according to the initial settings in Table 5, the genetic distances remain unchanged during the process, i.e. *d*_*AB*_=1,*d*_*AC*_=1 and *d*_*BC*_=1 hold. Hence, the distribution we are about to achieve can be calculated by Eq. (37).

37 $$ \begin{aligned} p(r,t) &= \int_{{t_{E}} = 0}^{+ \infty} {\int_{{t_{D}} = 0}^{{t_{E}}} {\int_{{d_{D}} = 0}^{0.5} {p({t_{E}},{t_{D}})}} \times p\left(\frac{{0.5}}{{{t_{D}}}}\right)} \\&\times p\left(\frac{{0.5}}{{{t_{D}}}}\right) \times p\left(\frac{{{d_{D}}}}{{{t_{E}} - {t_{D}}}}\right) \times p\left(\frac{{0.5 - {d_{D}}}}{{{t_{E}}}}\right){d_{d_{D}}}{d_{t_{D}}}{d_{t_{E}}} \end{aligned}  $$

The statistical measurements, i.e. mean and standard deviation, are compared in Table 6. The histograms of samples and numerical curves of *d*_*D*_ and *t*_*E*_ are pictured in Fig. 15c and d. It is shown that the two distributions are consistent within the acceptable error range. Therefore, Big Pulley can also give the right combinations of rates and node times, under the condition that the genetic distances among taxa are constant.

### 3. performance analysis of operators

This section provides the details of the results presented in *Performance comparison* section.

**3.1 Operator weights**

The weights on operators for the simulations when comparing efficiency are listed in Table 7. Although how to assign weights to achieve better performance is not studied in this paper, we maintain the percentage of weights on three operator class in Category and Cons configurations. But we modified some weights on the operators inside the same class, and we assigned different weights for different data sets.

**Table 7:** Operator weights in MCMC chains

Operator class	Name	Simulated data		Anolis		RSV2		HIV-1		Primates	
		Cons	Category	Cons	Category	Cons	Category	Cons	Category	Cons	Category
rates times	ConstantDistance Operator	0.2170	-	0.2248	-	0.2228	-	0.2228	-	0.2402	-
	Rate Normal Operators^1^	0.1302	0.2604	0.1349	0.2698	0.1337	0.2674	0.1337	0.2674	0.1441	0.2882
	UcldStdev Scale Operator^2^	0.0260	0.0260	0.0270	0.0270	0.0267	0.0267	0.0267	0.0267	0.0288	0.0288
	UcldMean Scale Operator	-	-	-	-	0.0089	0.0089	0.0089	0.0089	-	-
	UcldMean Tree UpperDown Operator	-	-	-	-	0.0267	0.0267	0.0267	0.0267	-	-
	InternalNodeTime Scale Operator	0.0174	0.0260	0.0180	0.0270	0.0178	0.0267	0.0178	0.0267	0.0192	0.0288
	RootAge Scale Operator	0.0174	0.0260	0.0180	0.0270	0.0178	0.0267	0.0178	0.0267	0.0192	0.0288
	AllNodeTimes Uniform Operator	0.1910	0.2604	0.1978	0.2698	0.1961	0.2674	0.1961	0.2674	0.2113	0.2882
Tree	SubtreeSlide Operator	0.1302	0.1302	0.0989	0.0989	0.1337	0.1337	0.1337	0.1337	0.441	0.1441
	NarrowExchange Operator	0.1302	0.1302	0.0989	0.0989	0.1337	0.1337	0.1337	0.1337	0.0480	0.0480
	WideExchange Operator	0.0434	0.0434	0.0270	0.0270	0.0267	0.0267	0.0267	0.0267	0.0480	0.0480
	WilsonBalding Operator	0.0434	0.0434	0.0270	0.0270	0.0267	0.0267	0.0267	0.0267	0.0480	0.0480
	BirthRate Scale Operator	0.0434	0.0434	0.0629	0.0629	-	-	-	-	0.0288	0.0288
	DeathRate Scale Operator	-	-	0.0629	0.0629	-	-	-	-	-	-
	PopulationSize Scale Operator	-	-	-	-	0.0267	0.0267	0.0267	0.0267	-	-
Substitution model	Kappa Scale Operator	0.0087	0.0087	0.0009	0.0009	0.0009	0.0009	0.0009	0.0009	0.0192	0.0192
	Frequencies DeltaExchange Operator	0.0017	0.0017	0.0009	0.0009	0.0009	0.0009	0.0009	0.0009	0.0010	0.0010

Note

1: Random walk operator and Swap operator in Cons configuration, Random walk operator, Scale operator and Swap operator in Category configuration.

2: The operator introduced in Appendix section 4 is used in Cons configuration, a Scale operator is used in Category configuration.

-: The parameter is not sampled and no operator is assigned.

**3.2 Simulated data sets**

**Fig. 16:**
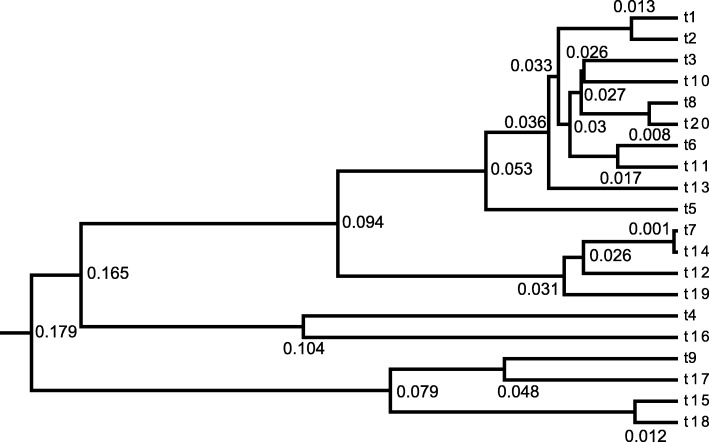
The tree used to simulate sequence alignment. The taxa are denoted by t1 to t20. The divergence times are drawn near the node

We simulated two sets of sequence alignment on the same tree with 20 taxa that is shown in Fig. 16. We used HKY model as substitution model with *κ*=2.4751, and the base frequencies are *π*=(0.21930.22680.30070.2531). In the uncorrelated relaxed clock model, the standard deviation of the branch rates (Ucldstdev) is 0.1803. The models and prior distributions are the same as is described in Fig. 1.

**Fig. 17:**
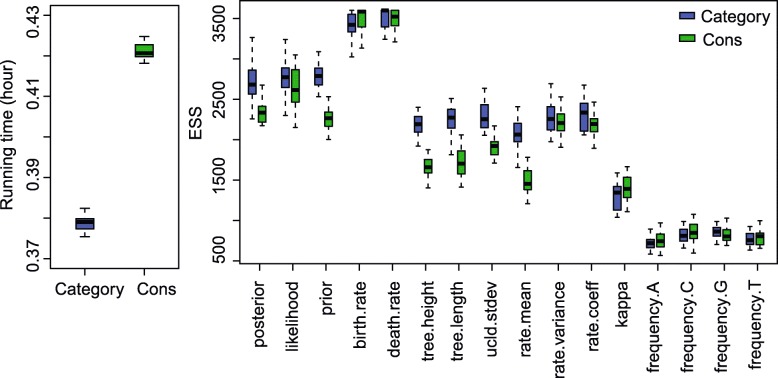
Running time and ESS using Anolis data

**3.3 Efficiency measured by ESS per hour**

**Fig. 18:**
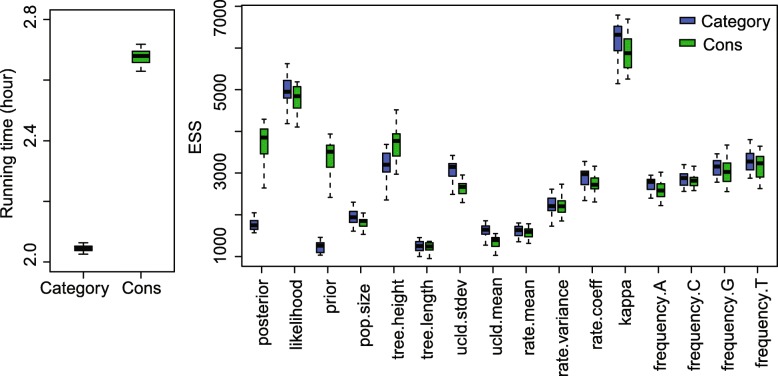
Running time and ESS using RSV2 data

**Fig. 19:**
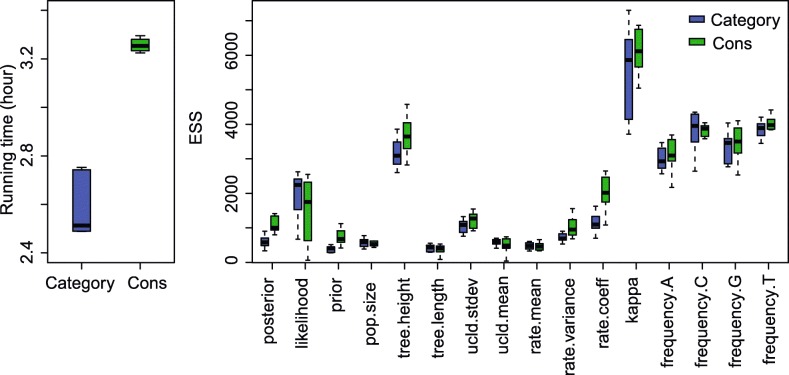
Running time and ESS using HIV-1 data

**Fig. 20:**
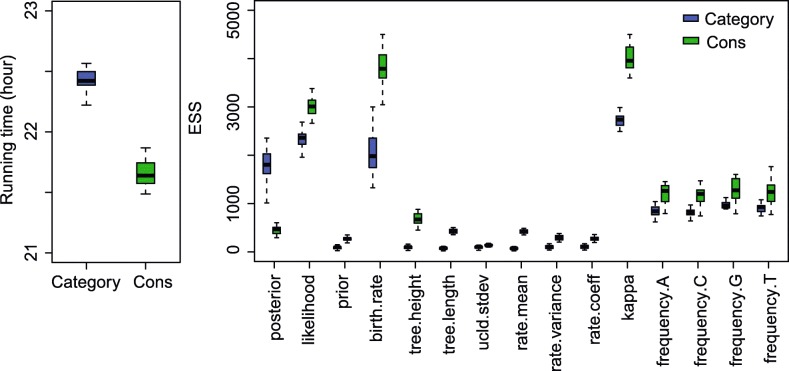
Running time and ESS using primates data

**Fig. 21:**
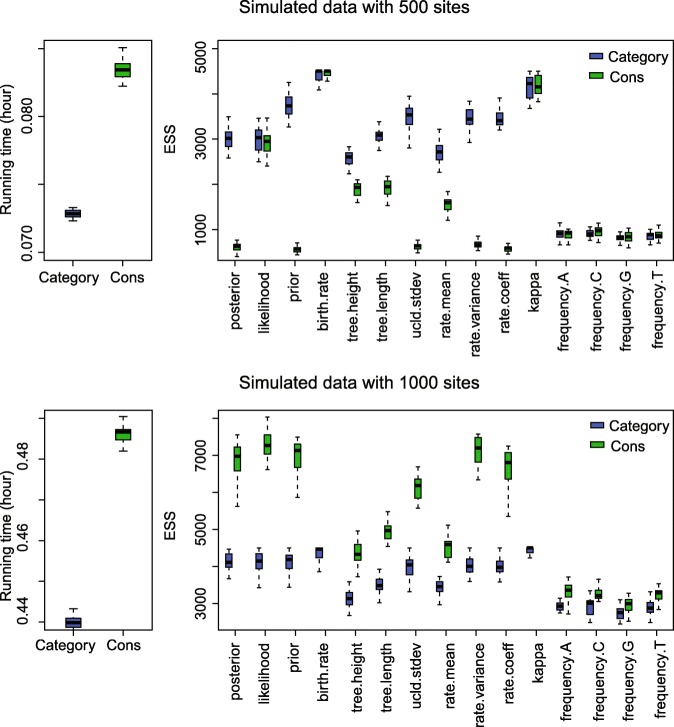
Running time and ESS using simulated data

Since we compare the efficiency based on ESS per hour using two configurations, i.e. Category and Cons, the ratio of ESS per hour is calculated by a random simulation in the two configurations, as is shown in Fig. 3. Then Table 2 lists the average running time and ESS of particular parameters in the simulations using different data sets. Here, we present the detailed running time and ESS of the simulations, which can be seen in Figs. 17, 18, 19, 20, and 21. Overall, we conclude that the proposed operators are able to provide better performance, because the figures suggest that Cons configuration requires less running time and have larger ESS for most parameters in most simulations. Especially, for those poorly estimated parameters in Category configuration, the improvement is more obvious. For data sets such as primates and simulated data with 500 sites, the running time is slightly larger in Cons configuration, but the ESS are much larger, which makes it acceptable to reduce the MCMC chain length and get the same performance.

**Fig. 22:**
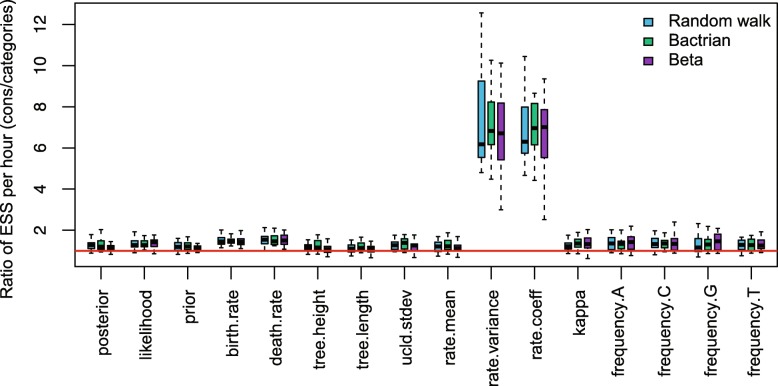
Efficiency comparison of proposals using Anolis data

**Fig. 23:**
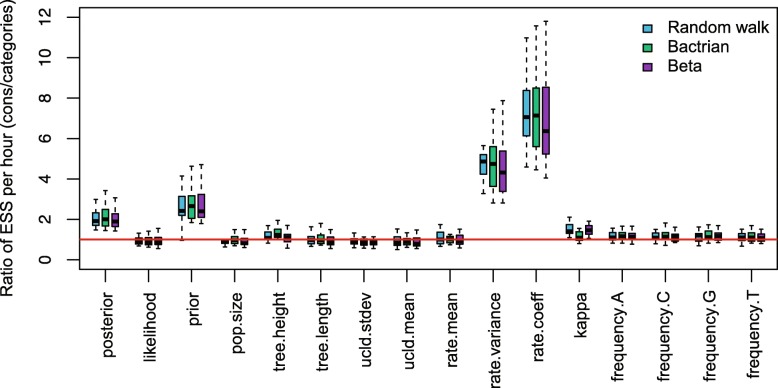
Efficiency comparison of proposals using RSV2 data

**Fig. 24:**
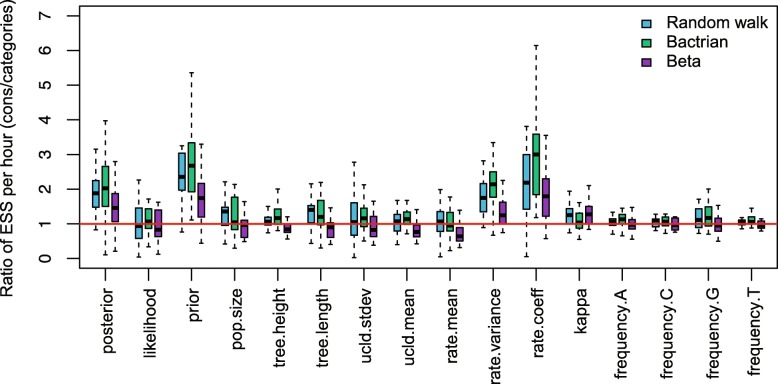
Efficiency comparison of proposals using HIV-1 data

**3.4 Efficiency measured by proposals**

The operators introduced in the paper utilise a random walk proposal for the new node time, which draws a random number from a uniform distribution and moves the node uniformly on the branch. However, others proposals, such as a Bactrian proposal [48] and a Beta proposal [49], assign a specific distribution on the new node time so that it is more probable to move to a certain height on the branch, either far away from or close to its original position. This section applied Random walk proposal (the operators in this paper), Bactrian proposal and Beta proposal to the three data sets, and the results are compared to those using Category configuration.

The comparisons are shown in Figs. 22, 23, and 24. It is indicated that Beta proposal achieved worst performance in the three analysed data sets. The performance of the Constant Distance operator (Random walk) and Bactrian proposal achieved similar performance in RSV2 data set, while Bactrian proposal provided larger ESS per hour for most parameters in HIV-1 data set. Therefore, it still needs further investigation to demonstrate the effectiveness of different proposals when analysing various data sets. Our current implementation of the operators enables users to specify which proposal style will be used in Beast2 analysis.

### 4. ucldstdevScaleOperator: a scale operator on standard deviation

It should be noted that the proposed ConstantDistance operator parameterises branch rates as continuous random variables, instead of discrete rate categories as is used in current BEAST2 settings. In uncorrelated relaxed clock model, branch rates are assumed to have a lognormal prior distribution, where the real mean is fixed to 1 and the standard deviation (denoted by Ucldstdev) is usually sampled with a hyper prior such as gamma(*α*=0.5396,*β*=0.3819). When a new Ucldstdev is proposed in one state during MCMC sampling by normal operators, the probability of all rates change as well under the new log normal distribution. Therefore, the authors implemented a separate operator working on Ucldstdev, which is able to solve this problem properly.

The first step is to propose a new Ucldstdev by a scale operation, which multiplies current Ucldstdev by a random factor, as is shown in Eq. (38).

38 $$ Ucldstdev' = Ucldstdev \times {\text{scale}}  $$

where ${\text {scale = Factor + }}\left [ \xi \times (\frac {1}{{{\text {Factor}}}}{\text {- Factor}}) \right ]$ and *ξ* is a random variable from a *Uniform*(0,1),Factor is a user-defined parameter to specify how bold the proposal is.

**Fig. 25:**
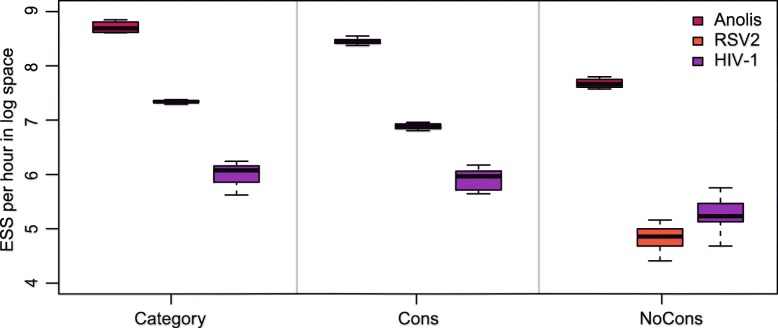
Efficiency comparison of clock standard deviation

Secondly, all the branch rates are proposed based on the new *Ucldstdev*^′^, given the probability of original *Ucldstdev*, which is calculated using Eq. (39).

39 $$ r_{i}' = icd{f_{stdev'}}\left[ {cd{f_{stdev}}({r_{i}})} \right]  $$

where the notations *cdf*(·) and *icdf*(·) represent the cumulative and inverse cumulative density function of log normal distribution. Because of the calculation of *cdf*(·) and *icdf*(·) for each branch rates, the "Cons" configuration requires more running time than "category", as is discusses in “Performance comparison” section. However, it is acceptable as ConstantDistance operator gives larger ESS.

Finally, it is important to return the corrected hastings ratio, since the proposal is associated with one random variable, *Ucldstdev* and (2*n*−1) branch rates. As is shown in Eq. (40), the ratio includes the scale operation and rates changing under the same probability.

40 $$ {{\mathbf{J}}_{Ucldstdev}} = \frac{1}{{scale}} \times \prod\limits_{i = 1}^{2n - 1} {\frac{{\partial icd{f_{Ucldstdev'}}[cd{f_{Ucldstdev}}({r_{i}})]}}{{\partial {r_{i}}}}}  $$

In the comparison of ESS for the clock standard deviation (denoted by ucld.stdev in Fig. 3), we specified a normal scale operator in “Category" configuration. In “Cons" configuration, the UcldstdevScaleOperator is used to sample the clock standard deviation of continuous rates. To avoid the concern that the difference between “Category" and “Cons" is a result of how rates are parameterised (i.e. discrete or continuous), we set another configuration where continuous rates are sampled without using the ConstantDistance operator (denoted by “NoCons" configuration). The weights of the operators in “NoCons" are the same as those in “Category" which is detailed in Table 7. We ran the analysis using the three real data sets (Anolis, RSV2 and HIV-1) and the comparison of ESS per hour between “Category”, “Cons" and “NoCons” is summarised in Fig. 25. The figure shows ESS per hour in *log*_10_ space of ucld.stdev in 20 independent MCMC chains. As can be seen, “Cons” configuration gives similar performance, comparing with “Category”. This indicates UcldstdevScaleOperator works properly on continuous rates. Moreover, ESS per hour is much larger in “Cons" than in “NoCons”, where both continuous rates are sampled. Therefore, the proposed operators contribute to the improved performance. However, we noticed that the rate parameterisation does have some mixing issues in MCMC chains. In the future, we will further investigate how to parameterise branch rates to get better performance when using the proposed operators.

## Data Availability

The source code of the proposed operator and the data sets analysed during the current study are available in the Github repository (https://github.com/Rong419/ConstantDistanceOperator.git).

## Abbreviations

MCMC: Markov chain Monte Carlo
ESS: effective sample sizes
HPD: highest posterior density
Category: Using the current operators in BEAST2 to sample discrete rate categories
Cons: Using the Constant Distance operator to sample continuous rates specified by an uncorrelated related clock model
